# Shift work and the risk of incident hospital-treated infections: quantifying the role of lifestyle factors

**DOI:** 10.1186/s12879-026-12979-3

**Published:** 2026-03-10

**Authors:** Ying Zhou, Yan Chen, Minzhi Xu, Hongyu Yan, Xiaoxv Yin

**Affiliations:** 1 Department of Social Medicine and Health Management, School of Public Health, Tongji Medical College, Huazhong University of Science and Technology, No.13 Hangkong Road, Wuhan, 430030 Hubei P.R. China; 2 Department of Geriatrics, Union Hospital, Tongji Medical College, Huazhong University of Science and Technology, Jiefang Avenue, Wuhan, Hubei 430022 China

**Keywords:** Shift work, Lifestyle, Hospital-treated infections, Cohort study

## Abstract

**Background:**

While the adverse effects of shift work on non-communicable diseases are widely recognized, research on its impact on infectious diseases is limited. We aim to investigate the association of shift work with hospital-treated infections through a large cohort, and to explore the mediating role of lifestyles in this association.

**Methods:**

This cohort study included employed workers from the UK Biobank. Work status was self-reported at baseline (2006–2010). A total of 866 types of hospital-treated infectious diseases were identified through hospital inpatient records. Lifestyle factors included smoking, alcohol consumption, body mass index, sedentary behavior, sleep duration, diet, and physical activity. We performed Cox proportional hazards regression models to examine the association of shift work with infections, and cause mediation analyses to explore the mediating role of lifestyles.

**Results:**

Of 266,450 employed workers, 45,406 (17.04%) reported working shifts. During a median follow-up of 12.47 years (IQR 11.55–13.25), 40,684 workers (15.27%) developed severe infections that required hospital treatment. Shift work was associated with a higher risk of infectious diseases (HR 1.10, 95% CI 1.07–1.13), and comparable risks were observed across different shift frequencies (sometimes: HR 1.09, 95% CI 1.05–1.13; usually/always: HR 1.10, 95% CI 1.07–1.14).

**Conclusions:**

In this cohort study, shift work was associated with higher risks of hospital-treated infections, with lifestyle variables mediating this association. It is recommended to consider shift work as an occupational hazard and implement individual-level and organizational-level interventions to mitigate vulnerability to infectious diseases.

**Supplementary information:**

The online version contains supplementary material available at 10.1186/s12879-026-12979-3.

## Introduction

With the rapid development of the economy and the prevalence of 24-hour service culture, shift work is becoming more and more common. [1, 2] Shift work enables the uninterrupted functioning of key industries and services at the societal level, but it may have a negative impact on the physical and mental health of workers. Current evidence suggests that shift work—generally encompassing work schedules outside the standard daytime hours (9 am to 5 pm), such as evening, night, or rotating shifts—is associated with various chronic non-communicable diseases such as type 2 diabetes [3, 4] and cardiovascular disease [5], which has sparked widespread discussion about developing measures to protect the health of shift workers. A detailed understanding of the susceptibility of shift work to adverse outcomes is needed to properly inform these efforts.

Infectious diseases remain a critical global health burden, contributing significantly to morbidity and mortality. [6] The emergence of the Coronavirus Disease 2019 further heightened the importance of identifying populations at increased risk of severe infectious outcomes and implementing targeted preventive measures. A previous observational study suggests that individuals engaged in non-standard working schedules, particularly shift workers, may be more susceptible to Coronavirus Disease 2019. [7] The association has been further supported by a recent systematic review and meta-analysis. [8] This observation raises the hypothesis that circadian disruption from shift work might increase susceptibility to a broader spectrum of infections. While prior research has explored links between shift patterns and infection rates, many studies rely on cross-sectional designs or relatively narrow assessment windows and focus on specific types of infections or selected occupational groups, which may complicate the establishment of clear temporal ordering and limit the characterization of overall infection risk. Most existing studies have primarily examined self-reported or outpatient-treated infections, which are often mild or self-managed. [9–12] By contrast, hospital-treated infections, defined as infections requiring inpatient care and recorded in hospital registers, represent clinically relevant outcomes with greater implications for healthcare utilization and population health. Furthermore, shift work is frequently associated with unhealthy lifestyle behaviors that may impair immune function and increase susceptibility to infectious diseases. [13, 14] However, the extent to which such modifiable lifestyle factors might mediate the relationship between shift work and infection risk remains insufficiently understood. Investigating these intermediary mechanisms could offer actionable insights for workplace and individual-level health interventions.

To address these important gaps, our study aimed to prospectively investigate the association of shift work with hospital-treated infections by using data from the UK Biobank, and further explore the mediating role of lifestyles between shift work and hospital-treated infections.

## Methods

### Study design and participants

The UK Biobank is a large-scale prospective cohort that collected baseline data of over 500,000 participants in Wales, England, and Scotland from March 2006 to October 2010. Data were obtained through verbal interviews, touchscreen questionnaires, biological samples, and physical measurements. The detailed introduction of the UK Biobank was presented elsewhere. [15, 16] Among a total of 502,401 participants at baseline, we excluded those with preexisting hospital-treated infections and those not in paid employment or self-employed. Preexisting hospital-treated infections were identified using hospital inpatient records, and participants whose first recorded infection occurred before or on the baseline assessment date were excluded to ensure that only incident infections during follow-up were analyzed. In the mediation study, we further excluded participants with missing data on lifestyle variables. Finally, 266,450 participants were included in the analysis of the association between shift work and hospital-treated infections, and 193,702 participants were included in the mediation analysis (Figure S1).

The UK Biobank obtained ethical approval from the North West Multi-Centre Research Ethical Committee with written informed consent from all participants.

### Assessment of shift work

The definitions of shift work and night shifts were explicitly presented to the participants during the baseline assessment via the touchscreen questionnaire. Shift work, as defined by the UK Biobank, is a work schedule that falls outside of the normal daytime working hours (9 am-5 pm). [17, 18] Shift work involves working in the afternoon, in the evening, at night, or rotating through these shifts. Night shifts refer to a work schedule that involves working during normal sleeping hours, for example, working from 12 am to 6 am.

At the time of the baseline survey, participants reported their employment status (in paid employment, self-employed, or other), whether their job involved alternative shifts (never/rarely, sometimes, usually, or always) and night shifts (never/rarely, sometimes, usually, or always), length of working per week (hours), time employed in current job (years), and whether their work involved heavy manual or physical labor, and walking or standing (never/rarely, sometimes, usually, or always). According to previously published studies, we classified workers who sometimes, usually, or always worked shifts as shift workers and those who never or rarely worked shifts as non-shift workers. [19] In this study, we examined the effects of shift work (yes or no), frequency of shift work (never/rarely, sometimes, usually/always), and type of shift work (night shift workers and non-night shift workers) on incident hospital-treated infections, respectively.

### Assessment of lifestyle factors

In this study, we evaluated seven lifestyle factors, including smoking status, alcohol consumption, body mass index (BMI), sedentary behavior, sleep duration, diet, and physical activity. BMI was objectively measured, while the other six lifestyle factors were assessed via self-reported touchscreen questionnaires at baseline. Smoking status was classified as current smokers and non-current smokers. [20] The combination of drinking frequency and estimated intake total was used to calculate alcohol consumption. [21, 22] One drink per day for females and two drinks per day for males was the cutting point for heavy alcohol intake (one drink is equivalent to eight grams of ethanol in the UK). [23] BMI was calculated as body weight divided by the square of height (kg/m^2^). Time spent on television was used as a proxy for sedentary behavior. [24] We regarded watching television for four hours or more per day as sedentary. Sleep duration was measured in hours, with seven to eight hours per day being a healthy sleep. [25] Diet was evaluated through a range of foods, namely, fruits, whole grains, refined grains, vegetables, fish, unprocessed red meats, and processed meats. [20] Based on the food frequency questionnaire, diet was categorized as healthy or unhealthy, with a healthy diet defined as meeting at least four of seven predefined dietary criteria. The cutting point for physical activity is 2.5 hours of moderate activity or 1.25 hours of vigorous activity per week, as recommended in the 2017 UK Physical Activity Guidelines. This measure captures total physical activity across all domains (including work). Table S1 provided a list of field codes and comprehensive definitions for all lifestyle variables.

### Ascertainment of hospital-treated infections

Diagnosis codes of all participants from hospital inpatient records in either the primary or secondary position were recorded in the UK Biobank and were coded using the International Classification of Disease version 10 (ICD-10). [26] We used a total of 866 ICD-10 codes to identify a wide range of infectious diseases. The outcome of our study was the infection with any of those infectious diseases. All the relevant ICD-10 codes were listed in Appendix S1. To facilitate clinically meaningful subtype analyses, hospital-treated infections were further classified according to the affected anatomical or organ systems into nine categories: upper respiratory tract, lower respiratory tract, gastrointestinal tract, skin and soft tissue, bloodstream, bone, joint and connective tissue, genitourinary system, heart and circulatory system, and neurological and eye infections. [27] The corresponding ICD-10 codes for each category are provided in Appendix S2. Follow-up duration was measured starting from the baseline date until the date of first infection diagnosis, death, end of follow-up, or loss to follow-up, whichever occurred first.

### Covariates

All covariates were assessed at baseline, including sociodemographic characteristics, health conditions, and work-related features: age (years), sex (male or female), education (college/university degree or other), ethnicity (White or others), Townsend deprivation index (a composite area-level measure of socioeconomic deprivation based on unemployment, non-car ownership, non-home ownership, and household overcrowding, with higher values indicating greater deprivation) [28], diabetes (yes or no), cardiovascular disease (yes or no), hypertension (yes or no), social isolation (yes or no), loneliness (yes or no), years working in current job (years), weekly working hours (hours), heavy manual or physical work (never/rarely, sometimes, usually, or always), and walking or standing at work (never/rarely, sometimes, usually, or always).

We identified patients with diabetes through algorithms that have been proven valid with 96% accuracy. [4, 29] Patients with cardiovascular disease and hypertension were identified through self-reported data and hospital admission ICD codes (Table S2). [22] Social isolation was determined by three dimensions: attendance in social or leisure activities, number of people living in the household, and frequency of family or friend visits. Loneliness was measured by two questions: frequency of confiding in close people and lonely feelings (Table S3). [30]

### Statistical analysis

Baseline characteristics of non-shift workers and shift workers were presented as number (percentage) for categorical variables, and mean (standard deviation [SD]) for continuous variables. χ^2^ tests and ANOVA were used to test statistical differences among groups.

We performed Cox proportional hazards models on the association of shift work, shift frequency, and shift type with the risk of overall hospital-treated infections and its subtypes. The cumulative incidence of hospital-treated infections by work schedule and shift frequency was presented using Kaplan-Meier curves. Three models were constructed. Model 1 adjusted for age and sex. Model 2 made additional adjustments for education, Townsend deprivation index, ethnicity, cardiovascular disease, diabetes, hypertension, loneliness, and isolation. Model 3 additionally adjusted for years working in current job, hours of work per week, heavy manual or physical work, or walking or standing at work. Missing values for covariates were handled using multiple imputation. Subgroup and interaction analyses stratified by age, sex, hours of work per week, heavy manual or physical work, and walking or standing at work were performed. In addition, exploratory subgroup analyses stratified by type of jobs were performed to assess potential heterogeneity across occupational categories, with job type categories defined in Appendix S3.

Cause mediation analyses based on a counterfactual framework were performed to assess the mediating effects of lifestyles on the association between shift work and hospital-treated infections. The causal diagram of hypothesized mediating effects and related assumptions was displayed in Figure S2. Counterfactual framework presents direct and indirect effects, and is more robust against the drawbacks of conventional adjustment-based mediation analyses. [31, 32] By integrating mediation and outcome models which made adjustment for covariates and other lifestyle factors outside the mediator, the total, direct, and indirect effects were computed. For the mediation models, natural indirect effects were estimated on the risk difference (RD) scale for the binary infection outcome. Using Quasi-Bayesian estimation with 1000 iterations, we calculated the 95% confidence intervals (CIs) for indirect effects. Mediation analyses were conducted in three steps. [33] Firstly, we used logistic or linear regression models with multivariate adjustments to evaluate the association between shift work and every lifestyle. Secondly, we estimated the associations between lifestyle factors and incident hospital-treated infections in adjusted Cox regression models, which served as preliminary analyses to identify candidate mediators. Thirdly, we performed mediation analyses using lifestyle characteristics that showed significant associations with shift work and infectious diseases. In addition to estimating the combined effects of lifestyle factors, we conducted a supplementary mediation analysis using a composite lifestyle score to estimate the overall mediating effect of lifestyles. The composite score was constructed by summing dichotomized lifestyle factors based on established criteria [34] and was included as a single mediator in a counterfactual-based mediation model.

Several sensitivity analyses were conducted to assess the robustness of our findings. Firstly, to minimize potential health selection bias, participants who developed infectious diseases < 1 year from baseline were excluded. Secondly, we further adjusted for frailty (assessed by walking speed, grip strength, weight loss, activity participation, and exhaustion), which may be a risk factor for infections. Thirdly, we adjusted non-mediating lifestyle factors (physical activity, dietary characteristics) in our models. Fourth, we excluded all cases with missing covariates values to reduce the bias caused by imputation. Fifth, to assess potential bias from exposure misclassification due to retirement or work status change, we performed sensitivity analyses restricting the cohort to participants aged under 60 years (or 55 years) at baseline. This restriction increases the probability that participants remained in the workforce during follow-up.

All the statistical analyses were conducted using R version 4.3.1. Two-sided *p* < 0.05 indicates statistical significance.

## Results

### Baseline characteristics of participants

Of 266,450 participants (47.9% male; mean [SD] age 52.8 [7.1] years), 45,406 (17.0%) were shift workers (55.0% male; mean [SD] age 51.9 [7.0] years), and the remaining were non-shift workers (46.5% male; mean [SD] age 52.9 [7.1] years). Compared to participants who did not work shifts, shift workers were younger, poorer, less educated, and were more likely to be male, non-white, feel lonely and isolated, do physical or manual labor, and walk or stand at work (Table 1).

**Table 1:** Baseline characteristics of the study participants according to their shift work status

Characteristics	Total (n = 266 450)	Current work schedule, No. (%)		*P * **value**
		Non-shift workers (n = 221 044)	Shift workers (n = 45 406)	
Age, years	52.8 (7.1)	52.9 (7.1)	51.9 (7.0)	<0.001
Sex				<0.001
Female	138748 (52.1)	118330 (53.5)	20418 (45.0)	
Male	127702 (47.9)	102714 (46.5)	24988 (55.0)	
Ethnicity				<0.001
White British	250283 (94.2)	209972 (95.3)	40311 (89.1)	
Other	15377 (5.8)	10454 (4.7)	4923 (10.9)	
Townsend deprivation index	−1.36 (3.00)	−1.52 (2.91)	−0.57 (3.27)	<0.001
Education				<0.001
College/University degree	100881 (38.4)	90706 (41.6)	10175 (22.8)	
Other	161805 (61.6)	127427 (58.4)	34378 (77.2)	
Diabetes				<0.001
No	257442 (96.6)	214009 (96.8)	43433 (95.7)	
Yes	9008 (3.4)	7035 (3.2)	1973 (4.3)	
Hypertension				<0.001
No	207120 (77.7)	172700 (78.1)	34420 (75.8)	
Yes	59330 (22.3)	48344 (21.9)	10986 (24.2)	
CVD				<0.001
No	257362 (96.6)	213786 (96.7)	43576 (96.0)	
Yes	9088 (3.4)	7258 (3.3)	1830 (4.0)	
Feeling lonely				<0.001
No	244248 (95.7)	204145 (96.0)	40103 (94.0)	
Yes	11004 (4.3)	8457 (4.0)	2547 (6.0)	
Socially isolated				<0.001
No	241129 (91.2)	201236 (91.6)	39893 (88.9)	
Yes	23411 (8.8)	18411 (8.4)	5000 (11.1)	
Years working in current job	12.92 (10.72)	12.89 (10.72)	13.05 (10.72)	0.005
Work hours a week, hours	35.29 (12.70)	34.74 (12.58)	37.99 (12.95)	<0.001
Walking or standing at work				<0.001
Never/rarely	94715 (35.6)	88093 (39.9)	6622 (14.6)	
Sometimes	81523 (30.6)	69224 (31.3)	12299 (27.1)	
Usually	39188 (14.7)	29381 (13.3)	9807 (21.6)	
Always	50799 (19.1)	34183 (15.5)	16616 (36.6)	
Heavy manual or physical labor at work				<0.001
Never/rarely	174183 (65.4)	158759 (71.9)	15424 (34.0)	
Sometimes	56794 (21.3)	39794 (18.0)	17000 (37.5)	
Usually	17887 (6.7)	11417 (5.2)	6470 (14.3)	
Always	17408 (6.5)	10957 (5.0)	6451 (14.2)	
Smoking status				<0.001
Non-current	237867 (89.5)	199415 (90.4)	38452 (85.0)	
Current	27878 (10.5)	21100 (9.6)	6778 (15.0)	
Physical activity				<0.001
Adequate	114342 (52.1)	92983 (50.5)	21359 (60.5)	
Inadequate	105114 (47.9)	91182 (49.5)	13932 (39.5)	
Alcohol drinking				<0.001
No heavy drinking	137599 (51.7)	112813 (51.1)	24786 (54.6)	
Heavy drinking	128678 (48.3)	108107 (48.9)	20571 (45.4)	
Diet				<0.001
Healthy	151249 (64.7)	127712 (65.6)	23537 (60.3)	
Unhealthy	82400 (35.3)	66911 (34.4)	15489 (39.7)	
Sleep duration, hours/day	7.05 (0.97)	7.07 (0.94)	6.94 (1.09)	<0.001
Sedentary behavior, hours				<0.001
<4	213221 (80.4)	179398 (81.5)	33823 (75.2)	
≥4	51952 (19.6)	40820 (18.5)	11132 (24.8)	
BMI, kg/m^2^	27.2 (4.7)	27.0 (4.6)	28.0 (4.9)	<0.001

Data are presented as mean (SD) for continuous variables and n (%) for categorical variables.

Abbreviations: BMI, body mass index; CVD, cardiovascular disease

### Shift work and incident hospital-treated infections

**Fig. 1:**
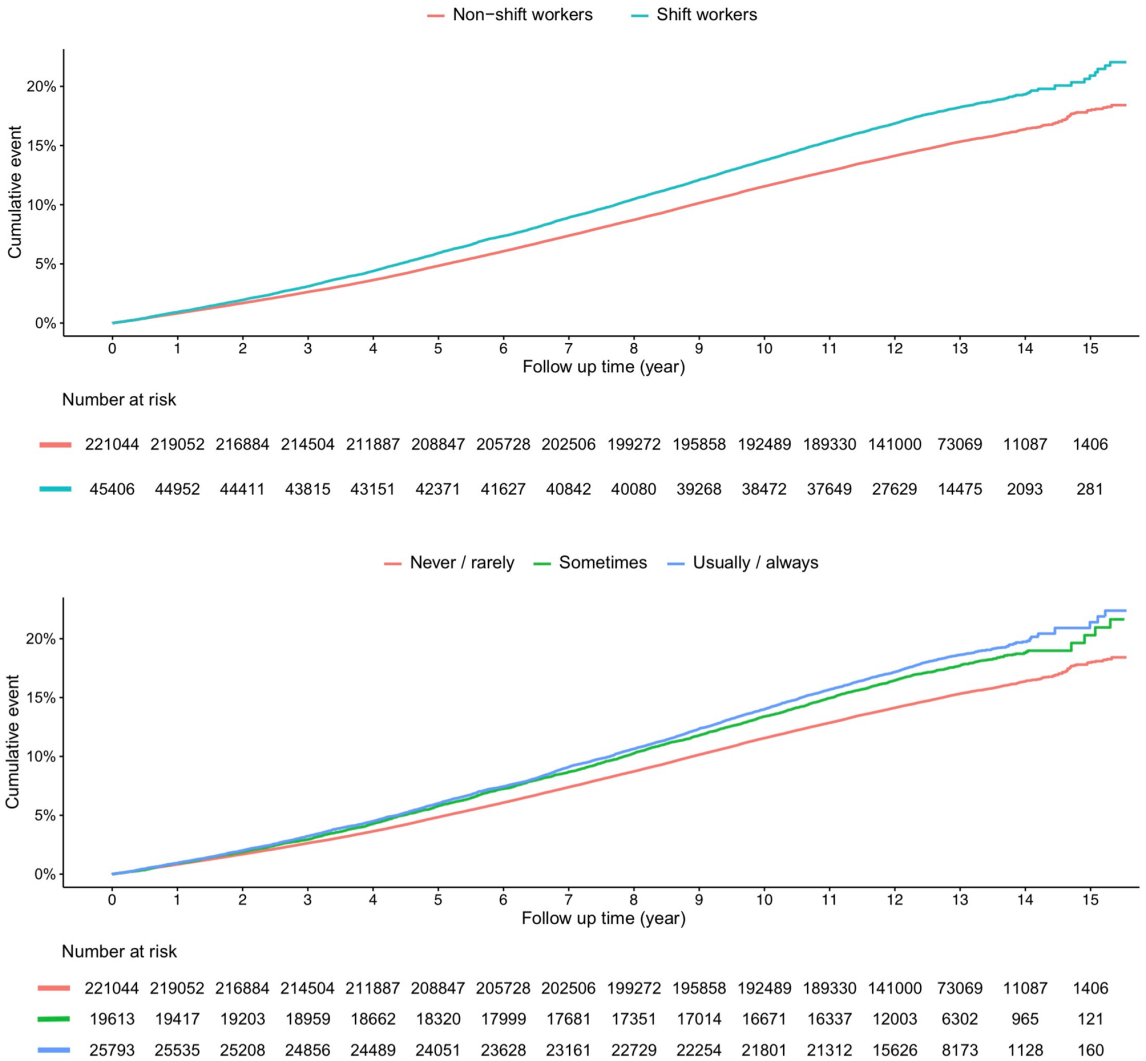
Kaplan-meier curves of time to primary outcome of hospital-treated infections by shift work and its frequency

The associations of shift work, shift work frequency, and night shift with the risk of incident hospital-treated infections were displayed in Table 2. A total of 40,684 workers (15.27%) developed infections during a median 12.47 years (IQR 11.55–13.25) of follow-up. Adjusting for age and sex, shift workers were associated with higher risk of infectious diseases (Hazard Ratio [HR] 1.26, 95% CI 1.23–1.29, *p* < 0.001). With further adjustment for more confounding factors, this risk was attenuated but still strongly significant (HR 1.10, 95% CI 1.07–1.13, *p* < 0.001). In terms of shift work frequency, an increased risk of incident hospital-treated infections was observed for both the “sometimes” (HR 1.09, 95% CI 1.05–1.13, *p* < 0.001) and “usually/always” (HR 1.10, 95% CI 1.07–1.14, *p* < 0.001) categories. However, no significant association was observed between night shifts and infection diseases among shift workers (HR 1.00, 95% CI 0.96–1.05, *p* = 0.877). The cumulative incidence of infections corresponding to work schedule and the frequency of shifts was presented in Fig. 1 using Kaplan-Meier curves. The associations between shift work and hospital-treated infection subtypes were presented in Table 3. The most common infection subtypes were gastrointestinal tract (*n* = 10,758), lower respiratory tract (*n* = 9,125), and genitourinary infections (*n* = 5,446). Shift work was associated with increased risks of lower respiratory tract, gastrointestinal tract, genitourinary infections, and skin and soft tissue infections.

**Table 2:** Associations of shift work and its frequency and type with incident hospital-treated infections

Characteristics		Rates per 1000 person-years	HR (95% CI)	*P * **value**
**Shift work**				
	Model 1			
	Non-shift work	12.61	1 [Reference]	
	Shift work	15.24	1.26 (1.23–1.29)	<0.001
	Model 2			
	Non-shift work	12.61	1 [Reference]	
	Shift work	15.24	1.14 (1.12–1.17)	<0.001
	Model 3			
	Non-shift work	12.61	1 [Reference]	
	Shift work	15.24	1.10 (1.07–1.13)	<0.001
	+Physical activity		1.11 (1.08–1.14)	<0.001
	+Dietary characteristics		1.09 (1.06–1.12)	<0.001
	+Smoking status		1.09 (1.06–1.12)	<0.001
	+Drinking habit		1.10 (1.07–1.13)	<0.001
	+Sleep duration		1.09 (1.06–1.12)	<0.001
	+Sedentary behavior		1.10 (1.07–1.13)	<0.001
	+BMI		1.08 (1.05–1.11)	<0.001
**Frequency of shift work**				
	Model 1			
	Never	12.61	1 [Reference]	
	Sometimes	14.80	1.21 (1.17–1.25)	<0.001
	Usually or always	15.58	1.30 (1.26–1.34)	<0.001
	Model 2			
	Never	12.61	1 [Reference]	
	Sometimes	14.80	1.13 (1.09–1.17)	<0.001
	Usually or always	15.58	1.15 (1.12–1.19)	<0.001
	Model 3			
	Never	12.61	1 [Reference]	
	Sometimes	14.80	1.09 (1.05–1.13)	<0.001
	Usually or always	15.58	1.10 (1.07–1.14)	<0.001
**Type of shift work**				
	Model 1			
	Shift but non-night shift workers	15.40	1 [Reference]	
	Night shift workers	15.09	1.02 (0.98–1.07)	0.309
	Model 2			
	Shift but non-night shift workers	15.40	1 [Reference]	
	Night shift workers	15.09	1.00 (0.96–1.05)	0.976
	Model 3			
	Shift but non-night shift workers	15.40	1 [Reference]	0.877
	Night shift workers	15.09	1.00 (0.96–1.05)	

Abbreviations: BMI, body mass index; HR, hazard ratio

Model 1, adjusted for age and sex; Model 2, adjusted for age, sex, education, Townsend deprivation index, ethnicity, cardiovascular disease, diabetes, hypertension, loneliness, and isolation; Model 3, adjusted for age, sex, education, Townsend deprivation index, ethnicity, cardiovascular disease, diabetes, hypertension, loneliness, isolation, years working in current job, hours of work per week, heavy manual or physical work, and walking or standing at work

**Table 3:** Associations of shift work with the risk of hospital-treated infection subtypes

Subtypes of infectious	Number of cases	HR (95% CI)	*P ***value**
Upper respiratory tract	1002	1.10 (0.93–1.31)	0.274
Lower respiratory tract	9125	1.16 (1.10–1.23)	<0.001
Gastrointestinal tract	10758	1.09 (1.03–1.15)	0.002
Genitourinary	5446	1.10 (1.02–1.19)	0.012
Bloodstream	2939	0.94 (0.84–1.05)	0.276
Heart and circulation	95	0.69 (0.36–1.33)	0.267
Bone, joint, and connective tissue	489	1.01 (0.79–1.29)	0.942
Neurological and eye	1395	1.00 (0.86–1.17)	0.978
Skin and soft tissue	5278	1.14 (1.05–1.22)	<0.001

Adjusted for age, sex, education, Townsend deprivation index, ethnicity, cardiovascular disease, diabetes, hypertension, loneliness, isolation, years working in current job, hours of work per week, heavy manual or physical work, and walking or standing at work

### Mediation analysis of lifestyle factors

Smoking status, drinking habit, sleep duration, sedentary behavior, and BMI were selected for mediation analyses since they were associated with both shift work and infectious diseases. Table 4 summarized the results of mediation analyses. Smoking, unhealthy drinking habit, unhealthy sleep duration, sedentary behavior, and BMI each showed evidence of natural indirect effects on the association between shift work and hospital-treated infections, with estimated indirect effects of 0.000737 (95% CI 0.000525-0.000982), 0.000084 (95% CI 0.000013-0.000164), 0.000801 (95% CI 0.000617-0.001017), 0.000091 (95% CI 0.000036-0.000162), and 0.001840 (95% CI 0.001606-0.002126), respectively. These indirect effects were estimated on the RD scale and represent absolute changes in the probability of hospital-treated infection mediated through each lifestyle factor. Detailed association estimates of shift work with every lifestyle and of lifestyle with incident hospital-treated infections were presented in Table S4 and Table S5. Mediation charts that showed the direct, indirect, and overall effects of each mediating lifestyle were displayed in Figure S3. A supplementary mediation analysis using a composite lifestyle score to estimate the overall mediating effect of lifestyles was conducted and was reported in Figure S4, with the total natural indirect effect estimated at 0.001493 (95% CI 0.001255-0.001750).

**Table 4:** Summary of mediation analyses

Characteristics	**Association with shift work** ^**a**^	**Association with incident hospital-treated infections** ^**b**^	**Indirect effects** ^**c**^ **(95% CI)**
Inadequate physical activity	Ø	+	NA
Unhealthy dietary characteristics	Ø	+	NA
Current smoking	+	+	0.000737 (0.000525-0.000982)^***^
Unhealthy drinking habit	−	−	0.000084 (0.000013-0.000164)^*^
Unhealthy sleep duration	+	+	0.000801 (0.000617-0.001017)^***^
Sedentary behavior	+	+	0.000091 (0.000036-0.000162)^***^
BMI	+	+	0.001840 (0.001606-0.002126)^***^

Abbreviations: +, positive association; −, negative association; Ø, no significant association; BMI, body mass index; NA, not available

^a^Summarized from Table S4 in Supplementary files; multiple linear or logistic regression models with the potential mediators as the dependent variables and shift work as the independent variable. Adjusted for each other and for age, sex, education, Townsend deprivation index, ethnicity, cardiovascular disease, diabetes, hypertension, loneliness, isolation, years working in current job, hours of work per week, heavy manual or physical work, and walking or standing at work

^b^Summarized from Table S5 in Supplementary files; Cox regression models with potential mediators as independent variables. Adjusted for each other and for shift work, age, sex, education, Townsend deprivation index, ethnicity, cardiovascular disease, diabetes, hypertension, loneliness, isolation, years working in current job, hours of work per week, heavy manual or physical work, and walking or standing at work

^c^Indirect effects are reported on the risk difference (RD) scale

^*^P < 0.05; ^***^p < 0.001

### Subgroup, interaction, and sensitivity analyses

The results of subgroup analyses stratified by age, sex, working hours, walking or standing, and physical or manual labor were presented in Table S6, while the results stratified by job types were shown in Table S7. The associations between shift work and hospital-treated infections were consistent across these groups. Significant interactions were observed with sex (*P*_interaction_ = 0.004), walking or standing at work (*P*_interaction_ = 0.020), and heavy manual or physical work (*P*_interaction_ = 0.020). The risk of developing infectious diseases was higher among females (HR 1.14, 95% CI 1.09–1.18), those who never or rarely walk or stand at work (HR 1.17, 95% CI 1.10–1.25), and those who never or rarely do heavy manual or physical labor (HR 1.14, 95% CI 1.10–1.19).

In analyses stratified by job type (Table S7), evidence of heterogeneity across occupational categories was observed. Shift work was associated with a significantly higher risk of hospital-treated infections among workers in healthcare and nursing (HR 1.18, 95% CI 1.02–1.37), public, business and service sectors (HR 1.17, 95% CI 1.03–1.33), and technology and administration (HR 1.19, 95% CI 1.07–1.33). In contrast, no statistically significant associations were observed among workers in manufacturing, construction and transportation (HR 1.00, 95% CI 0.88–1.15), or in agriculture, natural resources and environmental engineering (HR 0.86, 95% CI 0.60–1.25). Given that occupational information was available for only 26.9% of participants, these findings should be interpreted with caution.

The results of sensitivity analyses were available in Table S8. After excluding workers who developed incident hospital-treated infections < 1 year from baseline, excluding cases with missing values, excluding participants over 60 years (or 55 years) at baseline, further adjusted for frailty and for non-mediating lifestyle factors (physical activity, dietary characteristics), our findings remained robust.

## Discussion

In this population-based study of 266,450 participants, shift work was associated with a higher risk of hospital-treated infections, and comparable risks were observed across different shift frequencies. Among shift workers, no significant difference was found in the risk of hospital-treated infections between night-shift and non-night-shift workers. By examining the mediation effect, the association between shift work and hospital-treated infections was partially mediated by smoking status, drinking habit, sleep duration, sedentary behavior, and BMI.

Our study extends existing evidence on the association between shift work and infection risk. Previous studies have mainly examined mild infectious outcomes such as common colds or influenza-like illness, and have suggested that shift work-related factors, particularly sleep disruption, may increase general infection susceptibility. [9, 35] In contrast, our study focused specifically on hospital-treated infectious diseases, representing more severe, clinically ascertained outcomes captured through registry-based diagnoses. By examining a broad spectrum of infections in a large population-based cohort, our results demonstrate that the adverse effects of shift work extend beyond mild infections to those requiring hospital care. The observed associations across major infection subtypes, including lower respiratory tract, gastrointestinal tract, genitourinary infections, and skin and soft tissue infections, are biologically plausible. Shift work and circadian disruption have been shown to impair both innate and adaptive immune responses, which may compromise systemic and local host defenses against a broad spectrum of pathogens. Prior studies have reported elevated risks of respiratory infections among shift workers, lending support to the role of immune dysregulation in infection susceptibility. [11] In addition, circadian misalignment may adversely affect mucosal immunity and physiological regulation in the gastrointestinal and genitourinary systems [36], while disrupted immune surveillance and inflammatory responses may increase susceptibility to soft tissue infections [37], thereby contributing to a higher likelihood of severe infections requiring hospital care. This complements prior UK Biobank studies linking shift work to COVID-19 risk [7, 10] and suggests that shift work is associated with increased susceptibility to severe infections across a range of pathogens, not limited to pandemic contexts.

Several plausible mechanisms may link shift work to greater susceptibility to infections. Generally, shift work is often accompanied by intense work and subjective stress, which leads to unhealthy lifestyles (such as lack of sleep, obesity, and smoking). [3] Unhealthy lifestyles may result in a variety of immune-lowering risk factors, such as chronic inflammation and chronic diseases, increasing the risk of infections. [14] Based on this hypothesis, we further analyzed the mediating role of various lifestyle factors in shift work and hospital-treated infections. Our findings revealed that the association of shift work with infectious diseases is mediated partially by smoking, alcohol consumption, sleep duration, sedentary behavior, and BMI. If causal, intervening in these unhealthy lifestyles could reduce the incidence of shift workers being admitted to hospital for infections. This suggests that individual-level interventions focusing on sleep hygiene, tobacco cessation, and weight management are essential components of health promotion for this population.

In the subgroup analyses, we found a stronger association between shift work and the risk of infections among females and shift workers engaged in non-manual labor. A stronger association among females might be attributed to the influence of their menstrual cycles. Shift work disturbs biological rhythms, and then affects hormonal regulation, including the menstrual cycle. [38] The irregularity of the menstrual cycle would disrupt the immune system, subsequently compromising its ability to mount an effective immune response against pathogens and increasing the risk of infection. Regarding shift workers engaged in non-manual labor, the reason for differential results may also be explained by the “healthy worker effect”. [4] Workers engaged in physically demanding occupations are likely to undergo rigorous health assessments during the selection process, ensuring that they meet the physical requirements of their jobs. In contrast, non-manual labor positions may not impose the same rigorous health assessments. In that case, workers who perform manual labor may be inherently healthier than those who perform non-manual labor, and therefore have a higher resistance to infectious diseases. Our results suggest that efforts to improve the health of shift workers should focus more on females and workers engaged in non-manual work to achieve a greater overall reduction in the risk of hospital-treated infections. These findings indicate the need for supportive workplace strategies aimed at mitigating circadian disruption and fatigue among vulnerable subgroups, including improved shift regularity, adequate recovery time between shifts, and targeted occupational health support.

To the best of our knowledge, this study included more than 260,000 workers and over 800 types of infections, making it probably the largest study to date on the association between shift work and a variety of severe infectious diseases. The ascertainment of hospital-treated infections was extracted from national health registries, so we were able to obtain complete follow-up data, which was independent of workers’ active willingness to participate. This study has limitations. First, shift work status and lifestyle factors were assessed only at baseline, whereas both shift work exposure and lifestyle behaviors are inherently time-varying and may change during the long follow-up period. Reliance on baseline-only measurements may therefore lead to exposure and mediator misclassification. Although nondifferential misclassification typically attenuates effect estimates in single exposure–outcome associations, within a mediation framework, misclassification of both the exposure and the mediator may interact, rendering the direction and magnitude of bias in the estimated indirect and direct effects complex and difficult to predict. Consequently, the specific mediation effect estimates derived from our analyses should be interpreted as preliminary and hypothesis-generating rather than as precise quantitative decompositions. Their primary value lies in highlighting lifestyle factors as potential pathways through which shift work may influence infection risk, while the accurate quantification of these pathways will require future studies incorporating repeated measurements of exposures and mediators over time. Second, shift work status was defined based on current employment at baseline, and more detailed information on shift characteristics, such as rotation patterns, cumulative exposure, or lifetime occupational history, was not available. As a result, heterogeneity in shift work exposure could not be fully captured, and some participants classified as non-shift workers might have had prior exposure to shift work, potentially leading to non-differential exposure misclassification and attenuation of the observed associations. Third, despite our efforts to account for many confounding factors, the influence of potential residual confounding factors could not be utterly removed, such as genetic factors or unmeasured diseases. Fourth, the workers recruited in this study were largely White British, so the generalizability of the findings to other populations is constrained.

## Conclusion

In conclusion, we found that shift work was associated with higher risks of hospital-treated infections. Our study supports the recognition of shift work as an occupational hazard and underscores the importance of implementing integrated organizational adjustments and personalized lifestyle interventions to mitigate the susceptibility to infectious diseases among shift workers.

## Electronic supplementary material

Below is the link to the electronic supplementary material.

Supplementary Material 1

## Data Availability

Data from UK Biobank are available on application at (http://www.ukbiobank.ac.uk/register-apply).

## Abbreviations

BMI: Body mass index
CI: Confidence intervals
HR: Hazard Ratio
ICD-10: International Classification of Disease version 10
SD: Standard deviation
RD: Risk difference

## References

[1] Vetter C, Devore EE, Wegrzyn LR, et al. Association between rotating night shift work and risk of coronary heart disease among women. JAMA. 2016;315:1726–34. 27115377 10.1001/jama.2016.4454 PMC5102147

[2] Wickwire EM, Geiger-Brown J, Scharf SM, et al. Shift work and shift work sleep disorder: clinical and organizational perspectives. Chest. 2017;151:1156–72. 28012806 10.1016/j.chest.2016.12.007 PMC6859247

[3] Shan Z, Li Y, Zong G, et al. Rotating night shift work and adherence to unhealthy lifestyle in predicting risk of type 2 diabetes: results from two large US cohorts of female nurses. BMJ. 2018;363:k 4641. 10.1136/bmj.k4641 PMC6247172 30464025

[4] Vetter C, Dashti HS, Lane JM, et al. Night shift work, genetic risk, and type 2 diabetes in the UK Biobank. Diabetes Care. 2018;41:762–69. 29440150 10.2337/dc17-1933 PMC5860836

[5] Torquati L, Mielke GI, Brown WJ, et al. Shift work and the risk of cardiovascular disease. A systematic review and meta-analysis including dose-response relationship. Scand J Work Environ Health. 2018;44:229–38. 29247501 10.5271/sjweh.3700

[6] Suff N, Waddington SN. The power of bioluminescence imaging in understanding host-pathogen interactions. Methods. 2017;127:69–78. 28694065 10.1016/j.ymeth.2017.07.001

[7] Maidstone R, Anderson SG, Ray DW, et al. Shift work is associated with positive COVID-19 status in hospitalised patients. Thorax. 2021;76:601–06. 33903187 10.1136/thoraxjnl-2020-216651 PMC8098298

[8] Loef B, Bosma E, van Kerkhof LWM, et al. Night-shift work and susceptibility to infectious diseases: a systematic review and meta-analysis. Scand J Work Environ Health. 2025;51:298–311. 40188463 10.5271/sjweh.4225 PMC12281634

[9] Prather AA, Carroll JE. Associations between sleep duration, shift work, and infectious illness in the United States: data from the National Health Interview Survey. Sleep Health. 2021;7:638–43. 34193397 10.1016/j.sleh.2021.05.004

[10] Fatima Y, Bucks RS, Mamun AA, et al. Shift work is associated with increased risk of COVID-19: findings from the UK Biobank cohort. J Sleep Res. 2021;30:e13326. 10.1111/jsr.13326 PMC8250353 33686714

[11] Loef B, van Baarle D, van der Beek AJ, et al. Shift work and respiratory infections in health-care workers. Am J Epidemiol. 2019;188:509–17. 30475977 10.1093/aje/kwy258 PMC6395171

[12] Loef B, Dollé MET, Proper KI, et al. Night-shift work is associated with increased susceptibility to SARS-CoV-2 infection. Chronobiol Int. 2022;39:1100–09. 35502475 10.1080/07420528.2022.2069031

[13] Tosoratto J, Tárraga López PJ, López-González ÁA, et al. Association of shift work, sociodemographic variables and healthy habits with obesity scales. Life (Basel). 2024;14:1503. 39598301 10.3390/life14111503 PMC11595592

[14] de Frel Dl, Atsma DE, Pijl H, et al. The impact of obesity and lifestyle on the immune system and susceptibility to infections such as COVID-19. Front Nutr. 2020;7:597600. 33330597 10.3389/fnut.2020.597600 PMC7711810

[15] Sudlow C, Gallacher J, Allen N, et al. UK Biobank: an open access resource for identifying the causes of a wide range of complex diseases of middle and old age. PLoS Med. 2015;12:e1001779. 10.1371/journal.pmed.1001779 PMC4380465 25826379

[16] Collins R. What makes UK Biobank special? Lancet. 2012;379:1173–74. 22463865 10.1016/S0140-6736(12)60404-8

[17] Kanki M, Nath AP, Xiang R, et al. Poor sleep and shift work associate with increased blood pressure and inflammation in UK Biobank participants. Nat Commun. 2023;14:7096. 37925459 10.1038/s41467-023-42758-6 PMC10625529

[18] Wang N, Sun Y, Zhang H, et al. Long-term night shift work is associated with the risk of atrial fibrillation and coronary heart disease. Eur Heart J. 2021;42:4180–88. 34374755 10.1093/eurheartj/ehab505

[19] Xu M, Yin X, Gong Y. Lifestyle factors in the association of shift work and depression and anxiety. JAMA Netw Open. 2023;6:e2328798. 10.1001/jamanetworkopen.2023.28798 PMC10425829 37578795

[20] Lourida I, Hannon E, Littlejohns TJ, et al. Association of lifestyle and genetic risk with incidence of dementia. JAMA. 2019;322:430–37. 31302669 10.1001/jama.2019.9879 PMC6628594

[21] Bradbury KE, Murphy N, Key TJ. Diet and colorectal cancer in UK Biobank: a prospective study. Int J Epidemiol. 2020;49:246–58. 30993317 10.1093/ije/dyz064 PMC7124508

[22] Han H, Cao Y, Feng C, et al. Association of a healthy lifestyle with all-cause and cause-specific mortality among individuals with type 2 diabetes: a prospective study in UK Biobank. Diabetes Care. 2022;45:319–29. 34857534 10.2337/dc21-1512

[23] Zhang Y-B, Chen C, Pan X-F, et al. Associations of healthy lifestyle and socioeconomic status with mortality and incident cardiovascular disease: two prospective cohort studies. BMJ. 2021;373:n604. 10.1136/bmj.n604 PMC8044922 33853828

[24] Wang B, Wang N, Sun Y, et al. Association of combined healthy lifestyle factors with incident dementia in patients with type 2 diabetes. Neurology. 2022;99:e2336–45. 10.1212/WNL.0000000000201231 36104282

[25] van Oort S, Jwj B, van Ballegooijen Aj, et al. Association of cardiovascular risk factors and lifestyle behaviors with hypertension: a mendelian randomization study. Hypertension. 2020;76:1971–79. 33131310 10.1161/HYPERTENSIONAHA.120.15761

[26] Ronaldson A, Arias de la Torre J, Sima R, et al. Prospective associations between depression and risk of hospitalisation for infection: findings from the UK Biobank. Brain Behav Immun. 2022;102:292–98. 35218891 10.1016/j.bbi.2022.02.023 PMC11924240

[27] Zheng J, Shi W, Yang Q, et al. Hospital-treated infectious diseases, infection burden and risk of parkinson disease: an observational and Mendelian randomization study. Brain Behav Immun. 2024;120:352–59. 38897329 10.1016/j.bbi.2024.06.016

[28] Townsend P, Phillimore P, Beattie A. Health and deprivation: inequality and the North. Routledge; 1988.

[29] Eastwood SV, Mathur R, Atkinson M, et al. Algorithms for the capture and adjudication of prevalent and incident diabetes in UK Biobank. PLoS ONE. 2016;11:e0162388. 10.1371/journal.pone.0162388 PMC5025160 27631769

[30] Elovainio M, Komulainen K, Sipilä PN, et al. Association of social isolation and loneliness with risk of incident hospital-treated infections: an analysis of data from the UK Biobank and Finnish health and social support studies. Lancet Public Health. 2023;8:e109–18. 10.1016/S2468-2667(22)00253-5 PMC9879771 36669514

[31] Richiardi L, Bellocco R, Zugna D. Mediation analysis in epidemiology: methods, interpretation and bias. Int J Epidemiol. 2013;42:1511–19. 24019424 10.1093/ije/dyt127

[32] Ho FK, Celis-Morales C, Gray SR, et al. Association and pathways between shift work and cardiovascular disease: a prospective cohort study of 238 661 participants from UK Biobank. Int J Epidemiol. 2022;51:579–90. 34414428 10.1093/ije/dyab144 PMC9082805

[33] Geng T, Zhu K, Lu Q, et al. Healthy lifestyle behaviors, mediating biomarkers, and risk of microvascular complications among individuals with type 2 diabetes: a cohort study. PLoS Med. 2023;20:e1004135. 10.1371/journal.pmed.1004135 PMC9831321 36626356

[34] Yang G, Cao X, Li X, et al. Association of unhealthy lifestyle and childhood adversity with acceleration of aging among UK Biobank participants. JAMA Netw Open. 2022;5:e2230690. 10.1001/jamanetworkopen.2022.30690 PMC9449787 36066889

[35] Loef B, van der Beek AJ, Hulsegge G, et al. The mediating role of sleep, physical activity, and diet in the association between shift work and respiratory infections. Scand J Work Environ Health. 2020;46:516–24. 32255192 10.5271/sjweh.3896 PMC7737798

[36] Cheng L, Wang X, Wang Q, et al. Circadian rhythm disturbance impairs intestinal mucus barrier and immune microenvironment via sebacic acid-mediated gut dysbiosis. Microbiol Res. 2026;303:128375. 41175695 10.1016/j.micres.2025.128375

[37] Tsujihana K, Tanegashima K, Santo Y, et al. Circadian protection against bacterial skin infection by epidermal CXCL14-mediated innate immunity. Proc Natl Acad Sci USA. 2022;119:e2116027119. 10.1073/pnas.2116027119 PMC9231475 35704759

[38] Lawson CC, Whelan EA, Lividoti Hibert EN, et al. Rotating shift work and menstrual cycle characteristics. Epidemiology. 2011;22:305–12. 21364464 10.1097/EDE.0b013e3182130016 PMC5303197

