# Supplementary material for: Shift work and the risk of incident hospital-treated infections: quantifying the role of lifestyle factors
Source: BMC Infect Dis. 2026 Mar 10;26:775. doi: 10.1186/s12879-026-12979-3 (PMC13088787; doi:10.1186/s12879-026-12979-3)
Supplement: Supplementary file 1 — Supplementary Material 1 [file 12879_2026_12979_MOESM1_ESM.docx]

**Shift work and the risk of incident hospital-treated infections: quantifying the role of lifestyle factors**

[Table S1. Assessment of lifestyle factors 2](#_Toc165113317)

[Table S2. Definitions of hypertension and cardiovascular disease 4](#_Toc165113318)

[Table S3. Definitions of loneliness and social isolation 5](#_Toc165113319)

[Table S4. Associations between shift work and potential mediators 6](#_Toc165113320)

[Table S5. Association between potential mediators and incident hospital-treated infections and mediation proportions 7](#_Toc165113321)

[Table S6. Associations of shift work with incident hospital-treated infections by subgroups 8](#_Toc165113322)

[Table S7. Associations of shift work with incident hospital-treated infections by type of job subgroups 9](#_Toc165113323)

[Table S8. Sensitivity analyses of shift work and its frequency and type in relation to incident hospital-treated infections 10](#TableS8)

[Figure S1. Flowchart for the selection of the study population 14](#_Toc165113324)

[Figure S2. Causal diagram for the relationship between shift work (X), lifestyle factors (M), and incident hospital-treated infections (Y) 15](#_Toc165113325)

[Figure S3. Mediation effects of lifestyle factors on the association of shift work and incident hospital-treated infections 16](#_Toc165113326)

[Figure S4. Mediation effects of the composite lifestyle score on the association of shift work and incident hospital-treated infections 17](#FigureS4)

[Appendix S1. Supplemental outcome measures 18](#AppendixS1)

[Appendix S2. Classification of site-specific hospital-treated infectious diseases in the study on diagnosis level](#AppendixS2) [42](#AppendixS2)

[Appendix S3. The codes and classification of the participants’ jobs 44](#AppendixS3)

Table S1. Assessment of lifestyle factors

BMI was objectively measured, while the other six lifestyle factors were assessed via self-reported touchscreen questionnaires at baseline. (https://biobank.ndph.ox.ac.uk/showcase/). Details of the assessment of health behaviors were shown as follows:

| **Lifestyle variables** | **Definition** | **Field code** |
| --- | --- | --- |
| Smoking status | Smoking status was defined as current smokers and non-current smokers. | 20116 |
| Alcohol consumption | The average amount of daily alcohol intake was calculated according to previous UK Biobank research (1, 2). The status of alcohol consumption was defined by integrated information on drinking frequency and estimated intake total. The cutting point of alcohol consumption was no more than one drink/day for women and two drinks/day for men. One drink is measured as 8 grams of ethanol in the UK. | 20117, 1558, 1568, 1578, 1588, 1598, 1608, 5364, 4407, 4418, 4429, 4440, 4451, 4462 |
| Diet | Based on the food frequency questionnaire, a diet that satisfies at least four of the following seven criteria is defined as a healthy diet.  1. Processed meats: ≤ 1 serving per week  2. Refined grains: ≤1.5 servings per day  3. Whole grains: ≥ 3 servings per day  4. Fish: ≥2 servings per week  5. Vegetables: ≥ 3 servings per day  6. Unprocessed red meats: ≤ 1.5 servings per week  7. Fruits: ≥ 3 servings per day | 1289, 1299, 1309, 1319, 1329, 1339, 1349, 1369, 1379, 1389, 1438, 1448, 1458, 1468 |
| Physical activity | Physical activity was defined as whether a person met the 2017 UK Physical activity guidelines of 150 minutes of moderate activity per week or 75 minutes of vigorous activity. | 22035 |
| Sleep duration | Sleep duration was defined as hours spent on sleep (including naps) every 24 hours. Sleeping for 7–8 h/day was defined as healthy sleep. | 1160 |
| Sedentary behavior | Television watching time was used as the proxy for sedentary behavior. We considered watching television for 4 hours and above per day to be sedentary behavior. | 1070 |
| Body mass index (BMI) | BMI value is constructed from height and weight measured during the initial Assessment Centre visit. BMI was calculated as body weight divided by the square of height (kg/m^2^) | 21001 |

**References**

1. Han H, Cao Y, Feng C, Zheng Y, Dhana K, Zhu S, Shang C, Yuan C, Zong G. Association of a Healthy Lifestyle With All-Cause and Cause-Specific Mortality Among Individuals With Type 2 Diabetes: A Prospective Study in UK Biobank. Diabetes Care. 2022 Feb 1;45(2):319-329. doi: 10.2337/dc21-1512.

2. Bradbury KE, Murphy N, Key TJ. Diet and colorectal cancer in UK Biobank: a prospective study. Int J Epidemiol. 2020 Feb 1;49(1):246-258. doi: 10.1093/ije/dyz064.

Table S2. Definitions of hypertension and cardiovascular disease

**Hypertension**: The definition was described according to the 9th and 10th revisions of the International Classification of Diseases (ICD-9 and ICD-10) and self-reported data fields.

**Cardiovascular disease**: The definition was described according to the 9th and 10th revisions of the International Classification of Diseases (ICD-9 and ICD-10) and self-reported data fields.

|  | **ICD-9** | **ICD-10** | **Self-reported in UK Biobank (field code)** |
| --- | --- | --- | --- |
| Hypertension | 401-405 | I10-I13, I15, O10 | 6150 (4), 20002 (1065, 1072), 6153 (2), 6177 (2) |
| Cardiovascular diseases ^*^ | 410-414, 430-434, 436 | I20-I25, I60-I64 | 6150 (1, 2, 3), 20002 (1074, 1075, 1081, 1583, 1086, 1491) |

^*^Contains coronary heart disease and stroke.

Table S3. Definitions of loneliness and social isolation

|  | **Definition** | **Field code** |
| --- | --- | --- |
| Loneliness | Loneliness was assessed by asking two questions: “Do you often feel lonely?” (no, 0; yes, 1) and “How often are you able to confide in someone close to you?” (0, almost daily–once every few months; 1, never or almost never). We defined a person as lonely only if they responded positively to both questions. | 2020, 2110 |
| Social isolation | Participants who reported “living alone”, “friends and family visit less than once a month”, and “no participation in social activities at least weekly” were assigned 1 point each. Other cases are assigned 0 point. After adding up the points, every participant had a social score ranging from 0-3. The social isolation status of participants was divided into “isolated” (score ≥ 2), “not socially isolated” (score <2). | 709, 1031, 6160 |

Table S4. Associations between shift work and potential mediators

|  | β (95% CI) | *P* |
| --- | --- | --- |
| Inadequate physical activity^*^ | -0.02 (-0.04, 0.01) | 0.299 |
| Unhealthy dietary characteristics^*^ | -0.02 (-0.05, 0.01) | 0.114 |
| Current smoking^*^ | 0.17 (0.13, 0.22) | <0.001 |
| Unhealthy drinking habit^*^ | -0.04 (-0.06, -0.01) | <0.01 |
| Unhealthy sleep duration^*^ | 0.21 (0.19, 0.24) | <0.001 |
| Sedentary behavior^*^ | 0.07 (0.04, 0.10) | <0.001 |
| BMI | 0.47 (0.42, 0.53) | <0.001 |
| β, standardized regression coefficients; BMI, body mass index. | |  |
| ^*^ Binary variables modelled by logistic regressions; the exponentiation of β was odds ratio. | | |
| Adjusted for each other and for age, sex, education, Townsend deprivation index, ethnicity, cardiovascular disease, diabetes, hypertension, loneliness, isolation, years working in current job, hours of work per week, heavy manual or physical work, and walking or standing at work. | | |

Table S5. Association between potential mediators and incident hospital-treated infections and mediation proportions

|  | Outcome regressed by potential mediator | Mediation analysis | |
| --- | --- | --- | --- |
|  | HR (95% CI) | Indirect effects ^a^ (95% CI) | *P*_mediation_ |
| Inadequate physical activity | 1.04 (1.02-1.07) | NA | 0.380 |
| Unhealthy dietary characteristics | 1.05 (1.03-1.08) | NA | 0.180 |
| Current smoking | 1.41 (1.36-1.46) | 0.000737 (0.000525-0.000982) | <0.001 |
| Unhealthy drinking habit | 0.93 (0.90-0.95) | 0.000084 (0.000013-0.000164) | 0.016 |
| Unhealthy sleep duration | 1.14 (1.11-1.17) | 0.000801 (0.000617-0.001017) | <0.001 |
| Sedentary behavior | 1.06 (1.03-1.09) | 0.000091 (0.000036-0.000162) | <0.001 |
| BMI | 1.03 (1.03-1.03) | 0.001840 (0.001606-0.002126) | <0.001 |
| HR: hazard ratio; CI: confidence interval; BMI, body mass index; | | | |
| Adjusted for each other and for shift work, age, sex, education, Townsend deprivation index, ethnicity, cardiovascular disease, diabetes, hypertension, loneliness, isolation, years working in current job, hours of work per week, heavy manual or physical work, and walking or standing at work.  ^a^ Indirect effects are reported on the risk difference (RD) scale. | | | |

Table S6. Associations of shift work with incident hospital-treated infections by subgroups

|  | HR (95% CI) | *P* _interaction_ |
| --- | --- | --- |
| **Age** |  | 0.379 |
| ≤50 | 1.09 (1.04- 1.14) |  |
| > 50 | 1.09 (1.05-1.12) |  |
| **Sex** |  | 0.004 |
| Female | 1.14 (1.09-1.18) |  |
| Male | 1.07 (1.04-1.11) |  |
| **Hours of work per week** |  | 0.360 |
| ≤40 | 1.09 (1.06-1.13) |  |
| > 40 | 1.12 (1.06-1.17) |  |
| **Walking or standing at work** | | 0.020 |
| Never or rarely | 1.17 (1.10-1.25) |  |
| Sometimes or more | 1.09 (1.06-1.12) |  |
| **Heavy manual or physical work** | | 0.020 |
| Never or rarely | 1.14 (1.10-1.19) |  |
| Sometimes or more | 1.07 (1.03-1.10) |  |
| HR: hazard ratio; CI: confidence interval | | |
| Adjusted for age, sex, education, Townsend deprivation index, ethnicity, cardiovascular disease, diabetes, hypertension, loneliness, isolation, years working in current job, hours of work per week, heavy manual or physical work, and walking or standing at work. Each group adjusted for the other covariates except itself. | | |

**Table S7. Associations of shift work with incident hospital-treated infections by type of job subgroups**

|  | HR (95% CI) |
| --- | --- |
| **Type of Jobs** |  |
| Healthcare and Nursing | 1.18 (1.02-1.37) |
| Agriculture, Natural Resources, and Environmental Engineering | 0.86 (0.60-1.25) |
| Public, Business, and Service Sectors | 1.17 (1.03-1.33) |
| Manufacturing, Construction, and Transportation | 1.00 (0.88-1.15) |
| Technology and Administration | 1.19 (1.07-1.33) |
| Adjusted for age, sex, education, Townsend deprivation index, ethnicity, cardiovascular disease, diabetes, hypertension, loneliness, isolation, years working in current job, hours of work per week, heavy manual or physical work, and walking or standing at work. | |

Table S8. Sensitivity analyses of shift work and its frequency and type in relation to incident hospital-treated infections

|  | HR (95% CI) | *P* |
| --- | --- | --- |
| Excluding participants with incident hospital-treated infections < 1 year from baseline ^a^ | | |
| Shift work |  |  |
| Non-shift work | Ref. |  |
| Shift work | 1.11 (1.08-1.14) | <0.001 |
| Frequency of shift work |  |  |
| Never | Ref. |  |
| Sometimes | 1.10 (1.06-1.14) | <0.001 |
| Usually or always | 1.11 (1.08-1.15) | <0.001 |
| Type of shift work |  |  |
| Shift but non-night shift workers | Ref. |  |
| Night shift workers | 1.00 (0.96-1.05) | 0.858 |
| Further adjusted for frailty ^b^ | | |
| Shift work |  |  |
| Non-shift work | Ref. |  |
| Shift work | 1.09 (1.06-1.12) | <0.001 |
| Frequency of shift work |  |  |
| Never | Ref. |  |
| Sometimes | 1.08 (1.04-1.12) | <0.001 |
| Usually or always | 1.09 (1.06-1.13) | <0.001 |
| Type of shift work |  |  |
| Shift but non-night shift workers | Ref. |  |
| Night shift workers | 1.02 (0.97-1.07) | 0.467 |
| Further adjustment for non-mediating lifestyle factors (physical activity, dietary characteristics) ^c^ | | |
| Shift work |  |  |
| Non-shift work | Ref. |  |
| Shift work | 1.11 (1.07-1.14) | <0.001 |
| Frequency of shift work |  |  |
| Never | Ref. |  |
| Sometimes | 1.09 (1.04-1.14) | <0.001 |
| Usually or always | 1.12 (1.07-1.16) | <0.001 |
| Type of shift work |  |  |
| Shift but non-night shift workers | Ref. |  |
| Night shift workers | 0.99 (0.93-1.05) | 0.677 |
| Excluding cases with missing values ^a^ | | |
| Shift work |  |  |
| Non-shift work | Ref. |  |
| Shift work | 1.11 (1.08-1.14) | <0.001 |
| Frequency of shift work |  |  |
| Never | Ref. |  |
| Sometimes | 1.11 (1.07-1.16) | <0.001 |
| Usually or always | 1.11 (1.07-1.15) | <0.001 |
| Type of shift work |  |  |
| Shift but non-night shift workers | Ref. |  |
| Night shift workers | 1.00 (0.96-1.05) | 0.916 |
| Excluding participants over 60 years ^a^ | | |
| Shift work |  |  |
| Non-shift work | Ref. |  |
| Shift work | 1.10 (1.06-1.13) | <0.001 |
| Frequency of shift work |  |  |
| Never | Ref. |  |
| Sometimes | 1.09 (1.05-1.14) | <0.001 |
| Usually or always | 1.10 (1.06-1.14) | <0.001 |
| Type of shift work |  |  |
| Shift but non-night shift workers | Ref. |  |
| Night shift workers | 1.02 (0.97-1.07) | 0.562 |
| Excluding participants over 55 years ^a^ |  |  |
| Shift work |  |  |
| Non-shift work | Ref. |  |
| Shift work | 1.09 (1.05-1.13) | <0.001 |
| Frequency of shift work |  |  |
| Never | Ref. |  |
| Sometimes | 1.08 (1.03-1.14) | 0.001 |
| Usually or always | 1.09 (1.04-1.14) | <0.001 |
| Type of shift work |  |  |
| Shift but non-night shift workers | Ref. |  |
| Night shift workers | 1.00 (0.95-1.06) | 0.914 |
| HR: hazard ratio; CI: confidence interval; | | |
| ^a^ Adjusted for age, sex, education, Townsend deprivation index, ethnicity, cardiovascular disease, diabetes, hypertension, loneliness, isolation, years working in current job, hours of work per week, heavy manual or physical work, and walking or standing at work. | | |
| ^b^ Adjusted for age, sex, education, Townsend deprivation index, ethnicity, cardiovascular disease, diabetes, hypertension, loneliness, isolation, years working in current job, hours of work per week, heavy manual or physical work, and walking or standing at work, and frailty. | | |
| ^c^ Adjusted for age, sex, education, Townsend deprivation index, ethnicity, cardiovascular disease, diabetes, hypertension, loneliness, isolation, years working in current job, hours of work per week, heavy manual or physical work, and walking or standing at work, physical activity, and dietary characteristics | | |

Figure S1. Flowchart for the selection of the study population


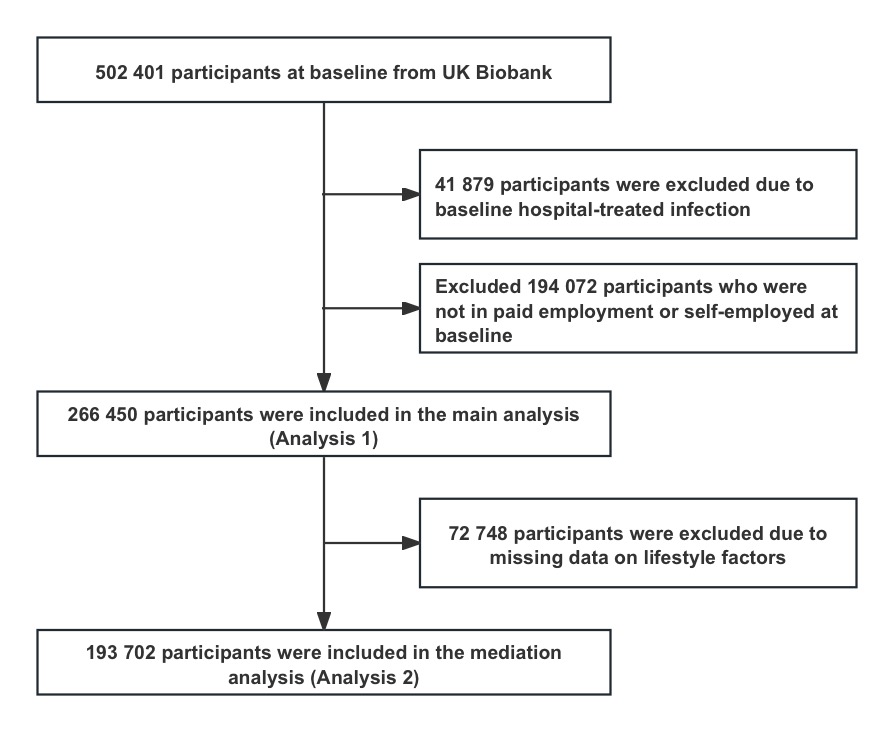


Figure S2. Causal diagram for the relationship between shift work (X), lifestyle factors (M), and incident hospital-treated infections (Y)

C and its solid arrows indicate measured confounders (age, sex, education, Townsend deprivation index, ethnicity, cardiovascular disease, diabetes, hypertension, loneliness, isolation, years working in current job, hours of work per week, walking or standing at work, and heavy manual or physical work), U_xy_ indicates unmeasured confounders of X-Y relation, U_my_ indicates unmeasured confounders of M-Y relation, U_xm_ indicates unmeasured confounders of X-M relation, U_c_ and its associated dashed arrows represent the identifying assumption that there are no measured or unmeasured confounders of the M-Y relation affected by X conditional on C.

Figure S3. Mediation effects of lifestyle factors on the association of shift work and incident hospital-treated infections


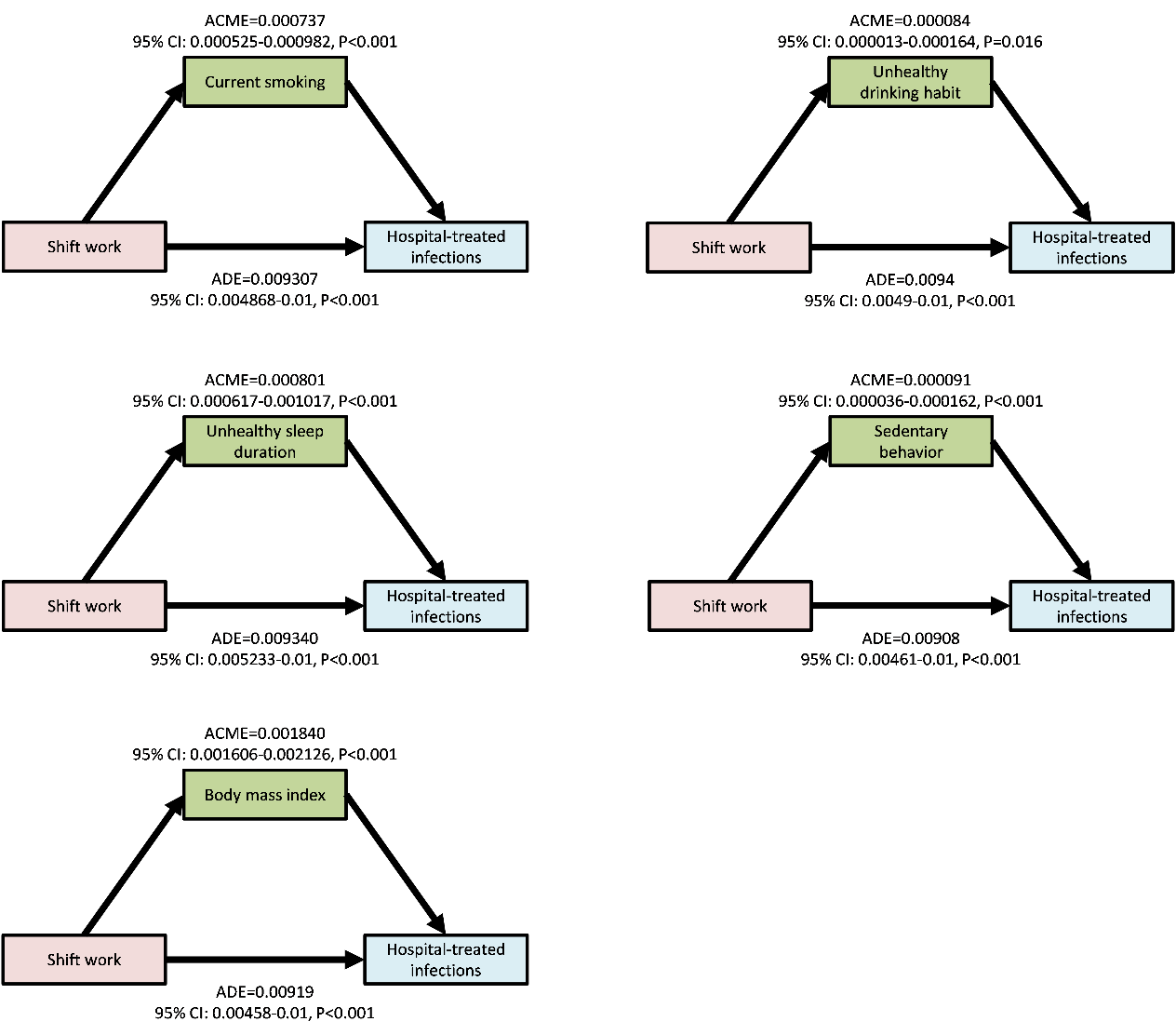


Abbreviations: ACME, average causal mediation effects; ADE, average direct effects; CI, confidence interval.

ACME are reported on the risk difference (RD) scale.

Figure S4. Mediation effects of the composite lifestyle score on the association of shift work and incident hospital-treated infections


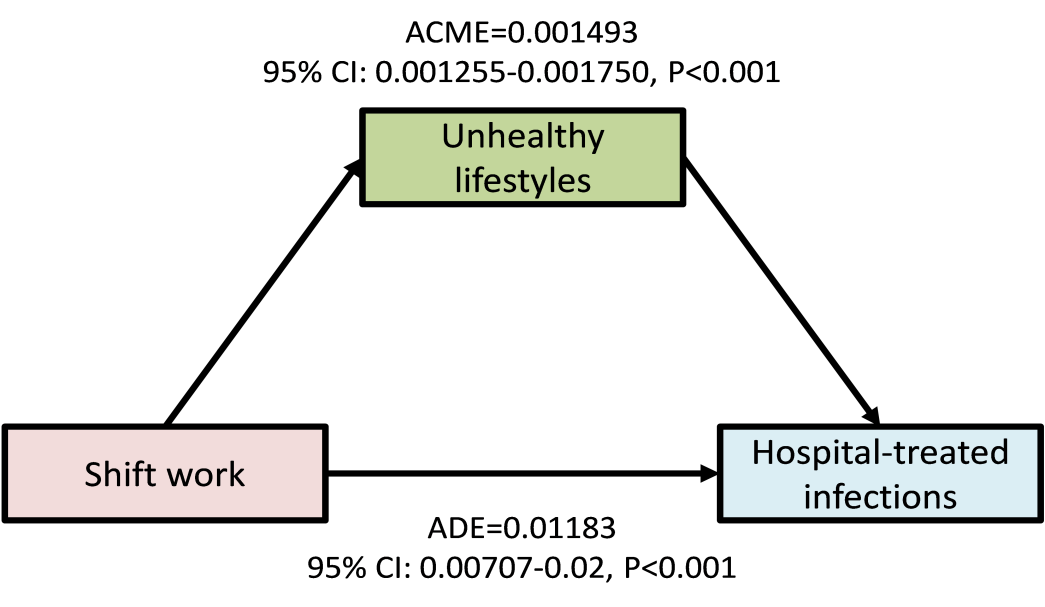


Abbreviations: ACME, average causal mediation effects; ADE, average direct effects; CI, confidence interval.

ACME are reported on the risk difference (RD) scale.

Appendix S1. Supplemental outcome measures

**Ascertainment for hospital-treated infections cases in UK Biobank**

| **ICD-10 code** | **ICD-10 name** |
| --- | --- |
| A00 | Cholera |
| A00.0 | Cholera due to Vibrio cholerae 01, biovar cholerae |
| A00.1 | Cholera due to Vibrio cholerae 01, biovar eltor |
| A00.9 | Cholera, unspecified |
| A01* | Typhoid and paratyphoid fevers |
| A02 | Other salmonella infections |
| A02.0 | Salmonella enteritis |
| A02.1 | Salmonella sepsis |
| A02.2 | Localized salmonella infections |
| A02.8 | Other specified salmonella infections |
| A02.9 | Salmonella infection, unspecified |
| A03 | Shigellosis |
| A03.0 | Shigellosis due to Shigella dysenteriae |
| A03.1 | Shigellosis due to Shigella flexneri |
| A03.2 | Shigellosis due to Shigella boydii |
| A03.3 | Shigellosis due to Shigella sonnei |
| A03.8 | Other shigellosis |
| A03.9 | Shigellosis, unspecified |
| A04 | Other bacterial intestinal infections |
| A04.0 | Enteropathogenic Escherichia coli infection |
| A04.1 | Enterotoxigenic Escherichia coli infection |
| A04.2 | Enteroinvasive Escherichia coli infection |
| A04.3 | Enterohaemorrhagic Escherichia coli infection |
| A04.4 | Other intestinal Escherichia coli infections |
| A04.5 | Campylobacter enteritis |
| A04.6 | Enteritis due to Yersinia enterocolitica |
| A04.7 | Enterocolitis due to Clostridium difficile |
| A04.8 | Other specified bacterial intestinal infections |
| A04.9 | Bacterial intestinal infection, unspecified |
| A05 | Other bacterial foodborne intoxications, not elsewhere classified |
| A05.0 | Foodborne staphylococcal intoxication |
| A05.1 | Botulism |
| A05.2 | Foodborne Clostridium perfringens [Clostridium welchii] intoxication |
| A05.3 | Foodborne Vibrio parahaemolyticus intoxication |
| A05.4 | Foodborne Bacillus cereus intoxication |
| A05.8 | Other specified bacterial foodborne intoxications |
| A05.9 | Bacterial foodborne intoxication, unspecified |
| A06 | Amoebiasis |
| A06.0 | Acute amoebic dysentery |
| A06.1 | Chronic intestinal amoebiasis |
| A06.2 | Amoebic nondysenteric colitis |
| A06.3 | Amoeboma of intestine |
| A06.4 | Amoebic liver abscess |
| A06.5 | Amoebic lung abscess |
| A06.6 | Amoebic brain abscess |
| A06.7 | Cutaneous amoebiasis |
| A06.8 | Amoebic infection of other sites |
| A06.9 | Amoebiasis, unspecified |
| A07* | Other protozoal intestinal diseases |
| A08.0 | Rotaviral enteritis |
| A08.1 | Acute gastroenteropathy due to Norwalk agent |
| A08.2 | Adenoviral enteritis |
| A08.3 | Other viral enteritis |
| A08.4 | Viral intestinal infection, unspecified |
| A08.5 | Other specified intestinal infections |
| A09* | Other gastroenteritis and colitis of infectious and unspecified origin |
| A15* | Respiratory tuberculosis, bacteriologically and histologically confirmed |
| A16* | Respiratory tuberculosis, not confirmed bacteriologically or histologically |
| A17 | Tuberculosis of nervous system |
| A17.0 | Tuberculous meningitis |
| A17.1 | Meningeal tuberculoma |
| A17.8 | Other tuberculosis of nervous system |
| A17.9 | Tuberculosis of nervous system, unspecified |
| A18* | Tuberculosis of other organs |
| A19* | Miliary tuberculosis |
| A20 | Plague |
| A20.0 | Bubonic plague |
| A20.1 | Cellulocutaneous plague |
| A20.2 | Pneumonic plague |
| A20.3 | Plague meningitis |
| A20.7 | Septicaemic plague |
| A20.8 | Other forms of plague |
| A20.9 | Plague, unspecified |
| A21 | Tularaemia |
| A21.0 | Ulceroglandular tularaemia |
| A21.1 | Oculoglandular tularaemia |
| A21.2 | Pulmonary tularaemia |
| A21.3 | Gastrointestinal tularaemia |
| A21.7 | Generalized tularaemia |
| A21.8 | Other forms of tularaemia |
| A21.9 | Tularaemia, unspecified |
| A22 | Anthrax |
| A22.0 | Cutaneous anthrax |
| A22.1 | Pulmonary anthrax |
| A22.2 | Gastrointestinal anthrax |
| A22.7 | Anthrax sepsis |
| A22.8 | Other forms of anthrax |
| A22.9 | Anthrax, unspecified |
| A23 | Brucellosis |
| A23.0 | Brucellosis due to Brucella melitensis |
| A23.1 | Brucellosis due to Brucella abortus |
| A23.2 | Brucellosis due to Brucella suis |
| A23.3 | Brucellosis due to Brucella canis |
| A23.8 | Other brucellosis |
| A23.9 | Brucellosis, unspecified |
| A24 | Glanders and melioidosis |
| A24.0 | Glanders |
| A24.1 | Acute and fulminating melioidosis |
| A24.2 | Subacute and chronic melioidosis |
| A24.3 | Other melioidosis |
| A25 | Rat-bite fevers |
| A25.0 | Spirillosis |
| A25.1 | Streptobacillosis |
| A25.9 | Rat-bite fever, unspecified |
| A26 | Erysipeloid |
| A26.0 | Cutaneous erysipeloid |
| A26.7 | Erysipelothrix sepsis |
| A26.8 | Other forms of erysipeloid |
| A26.9 | Erysipeloid, unspecified |
| A27* | Leptospirosis |
| A28 | Other zoonotic bacterial diseases, not elsewhere classified |
| A28.0 | Pasteurellosis |
| A28.1 | Cat-scratch disease |
| A28.2 | Extraintestinal yersiniosis |
| A28.8 | Other specified zoonotic bacterial diseases, not elsewhere classified |
| A28.9 | Zoonotic bacterial disease, unspecified |
| A30* | Leprosy [Hansen disease] |
| A31* | Infection due to other mycobacteria |
| A32 | Listeriosis |
| A32.0 | Cutaneous listeriosis |
| A32.1 | Listerial meningitis and meningoencephalitis |
| A32.7 | Listerial sepsis |
| A32.8 | Other forms of listeriosis |
| A32.9 | Listeriosis, unspecified |
| A33* | Tetanus neonatorum |
| A35* | Other tetanus |
| A36* | Diphtheria |
| A37* | Whooping cough |
| A38* | Scarlet fever |
| A39 | Meningococcal infection |
| A39.0 | Meningococcal meningitis |
| A39.1 | Waterhouse-Friderichsen syndrome |
| A39.2 | Acute meningococcaemia |
| A39.3 | Chronic meningococcaemia |
| A39.4 | Meningococcaemia, unspecified |
| A39.5 | Meningococcal heart disease |
| A39.8 | Other meningococcal infections |
| A39.9 | Meningococcal infection, unspecified |
| A40 | Streptococcal sepsis |
| A40.0 | Sepsis due to streptococcus, group A |
| A40.1 | Sepsis due to streptococcus, group B |
| A40.2 | Sepsis due to streptococcus, group D |
| A40.3 | Sepsis due to Streptococcus pneumoniae |
| A40.8 | Other streptococcal sepsis |
| A40.9 | Streptococcal sepsis, unspecified |
| A41 | Other sepsis |
| A41.0 | Sepsis due to Staphylococcus aureus |
| A41.1 | Sepsis due to other specified staphylococcus |
| A41.2 | Sepsis due to unspecified staphylococcus |
| A41.3 | Sepsis due to Haemophilus influenzae |
| A41.4 | Sepsis due to anaerobes |
| A41.5 | Sepsis due to other Gram-negative organisms |
| A41.8 | Other specified sepsis |
| A41.9 | Sepsis, unspecified |
| A42 | Actinomycosis |
| A42.0 | Pulmonary actinomycosis |
| A42.1 | Abdominal actinomycosis |
| A42.2 | Cervicofacial actinomycosis |
| A42.7 | Actinomycotic sepsis |
| A42.8 | Other forms of actinomycosis |
| A42.9 | Actinomycosis, unspecified |
| A43 | Nocardiosis |
| A43.0 | Pulmonary nocardiosis |
| A43.1 | Cutaneous nocardiosis |
| A43.8 | Other forms of nocardiosis |
| A44 | Bartonellosis |
| A44.0 | Systemic bartonellosis |
| A44.1 | Cutaneous and mucocutaneous bartonellosis |
| A44.8 | Other forms of bartonellosis |
| A44.9 | Bartonellosis, unspecified |
| A46* | Erysipelas |
| A48.0 | Gas gangrene |
| A48.1 | Legionnaires disease |
| A48.2 | Nonpneumonic Legionnaires disease [Pontiac fever] |
| A48.3 | Toxic shock syndrome |
| A48.4 | Brazilian purpuric fever |
| A48.8 | Other specified bacterial diseases |
| A49 | Bacterial infection of unspecified site |
| A49.0 | Staphylococcal infection, unspecified site |
| A49.1 | Streptococcal infection, unspecified site |
| A49.2 | Haemophilus influenzae infection, unspecified site |
| A49.3 | Mycoplasma infection, unspecified site |
| A49.8 | Other bacterial infections of unspecified site |
| A49.9 | Bacterial infection, unspecified |
| A50 | Congenital syphilis |
| A50.0 | Early congenital syphilis, symptomatic |
| A50.1 | Early congenital syphilis, latent |
| A50.2 | Early congenital syphilis, unspecified |
| A50.3 | Late congenital syphilitic oculopathy |
| A50.4 | Late congenital neurosyphilis [juvenile neurosyphilis] |
| A50.5 | Other late congenital syphilis, symptomatic |
| A50.6 | Late congenital syphilis, latent |
| A50.7 | Late congenital syphilis, unspecified |
| A50.9 | Congenital syphilis, unspecified |
| A51 | Early syphilis |
| A51.0 | Primary genital syphilis |
| A51.1 | Primary anal syphilis |
| A51.2 | Primary syphilis of other sites |
| A51.3 | Secondary syphilis of skin and mucous membranes |
| A51.4 | Other secondary syphilis |
| A51.5 | Early syphilis, latent |
| A51.9 | Early syphilis, unspecified |
| A52 | Late syphilis |
| A52.0 | Cardiovascular syphilis |
| A52.1 | Symptomatic neurosyphilis |
| A52.2 | Asymptomatic neurosyphilis |
| A52.3 | Neurosyphilis, unspecified |
| A52.7 | Other symptomatic late syphilis |
| A52.8 | Late syphilis, latent |
| A52.9 | Late syphilis, unspecified |
| A53* | Other and unspecified syphilis |
| A54 | Gonococcal infection |
| A54.0 | Gonococcal infection of lower genitourinary tract without periurethral or accessory gland abscess |
| A54.1 | Gonococcal infection of lower genitourinary tract with periurethral and accessory gland abscess |
| A54.2 | Gonococcal pelviperitonitis and other gonococcal genitourinary infections |
| A54.3 | Gonococcal infection of eye |
| A54.4 | Gonococcal infection of musculoskeletal system |
| A54.5 | Gonococcal pharyngitis |
| A54.6 | Gonococcal infection of anus and rectum |
| A54.8 | Other gonococcal infections |
| A54.9 | Gonococcal infection, unspecified |
| A55* | Chlamydial lymphogranuloma (venereum) |
| A56.0 | Chlamydial infection of lower genitourinary tract |
| A56.1 | Chlamydial infection of pelviperitoneum and other genitourinary organs |
| A56.2 | Chlamydial infection of genitourinary tract, unspecified |
| A56.3 | Chlamydial infection of anus and rectum |
| A56.4 | Chlamydial infection of pharynx |
| A56.8 | Sexually transmitted chlamydial infection of other sites |
| A57* | Chancroid |
| A58* | Granuloma inguinale |
| A59* | Trichomoniasis |
| A60* | Anogenital herpesviral [herpes simplex] infection |
| A63.0 | Anogenital (venereal) warts |
| A63.8 | Other specified predominantly sexually transmitted diseases |
| A64* | Unspecified sexually transmitted disease |
| A65* | Nonvenereal syphilis |
| A66* | Yaws |
| A67* | Pinta [carate] |
| A68* | Relapsing fevers |
| A69 | Other spirochaetal infections |
| A69.0 | Necrotizing ulcerative stomatitis |
| A69.1 | Other Vincent infections |
| A69.2 | Lyme disease |
| A69.8 | Other specified spirochaetal infections |
| A69.9 | Spirochaetal infection, unspecified |
| A70* | Chlamydia psittaci infection |
| A71* | Trachoma |
| A74.0 | Chlamydial conjunctivitis |
| A74.8 | Other chlamydial diseases |
| A74.9 | Chlamydial infection, unspecified |
| A75 | Typhus fever |
| A75.0 | Epidemic louse-borne typhus fever due to Rickettsia prowazekii |
| A75.1 | Recrudescent typhus [Brill disease] |
| A75.2 | Typhus fever due to Rickettsia typhi |
| A75.3 | Typhus fever due to Rickettsia tsutsugamushi |
| A75.9 | Typhus fever, unspecified |
| A77 | Spotted fever [tick-borne rickettsioses] |
| A77.0 | Spotted fever due to Rickettsia rickettsii |
| A77.1 | Spotted fever due to Rickettsia conorii |
| A77.2 | Spotted fever due to Rickettsia sibirica |
| A77.3 | Spotted fever due to Rickettsia australis |
| A77.8 | Other spotted fevers |
| A77.9 | Spotted fever, unspecified |
| A78* | Q fever |
| A79 | Other rickettsioses |
| A79.0 | Trench fever |
| A79.1 | Rickettsialpox due to Rickettsia akari |
| A79.8 | Other specified rickettsioses |
| A79.9 | Rickettsiosis, unspecified |
| A80* | Acute poliomyelitis |
| A81.1 | Subacute sclerosing panencephalitis |
| A81.2 | Progressive multifocal leukoencephalopathy |
| A83* | Mosquito-borne viral encephalitis |
| A84* | Tick-borne viral encephalitis |
| A85* | Other viral encephalitis, not elsewhere classified |
| A86* | Unspecified viral encephalitis |
| A87* | Viral meningitis |
| A88* | Other viral infections of central nervous system, not elsewhere classified |
| A89* | Unspecified viral infection of central nervous system |
| A90* | Dengue fever [classical dengue] |
| A91* | Dengue haemorrhagic fever |
| A92 | Other mosquito-borne viral fevers |
| A92.0 | Chikungunya virus disease |
| A92.1 | O'nyong-nyong fever |
| A92.2 | Venezuelan equine fever |
| A92.3 | West Nile virus infection |
| A92.4 | Rift Valley fever |
| A92.8 | Other specified mosquito-borne viral fevers |
| A92.9 | Mosquito-borne viral fever, unspecified |
| A93 | Other arthropod-borne viral fevers, not elsewhere classified |
| A93.0 | Oropouche virus disease |
| A93.1 | Sandfly fever |
| A93.2 | Colorado tick fever |
| A93.8 | Other specified arthropod-borne viral fevers |
| A94* | Unspecified arthropod-borne viral fever |
| A95* | Yellow fever |
| A96* | Arenaviral haemorrhagic fever |
| A97* | Dengue |
| A98 | Other viral haemorrhagic fevers, not elsewhere classified |
| A98.0 | Crimean-Congo haemorrhagic fever |
| A98.1 | Omsk haemorrhagic fever |
| A98.2 | Kyasanur Forest disease |
| A98.3 | Marburg virus disease |
| A98.4 | Ebola virus disease |
| A98.5 | Haemorrhagic fever with renal syndrome |
| A98.8 | Other specified viral haemorrhagic fevers |
| A99* | Unspecified viral haemorrhagic fever |
| B00 | Herpesviral [herpes simplex] infections |
| B00.0 | Eczema herpeticum |
| B00.1 | Herpesviral vesicular dermatitis |
| B00.2 | Herpesviral gingivostomatitis and pharyngotonsillitis |
| B00.3 | Herpesviral meningitis |
| B00.4 | Herpesviral encephalitis |
| B00.5 | Herpesviral ocular disease |
| B00.7 | Disseminated herpesviral disease |
| B00.8 | Other forms of herpesviral infection |
| B00.9 | Herpesviral infection, unspecified |
| B01 | Varicella [chickenpox] |
| B01.0 | Varicella meningitis |
| B01.1 | Varicella encephalitis |
| B01.2 | Varicella pneumonia |
| B01.8 | Varicella with other complications |
| B01.9 | Varicella without complication |
| B02 | Zoster [herpes zoster] |
| B02.0 | Zoster encephalitis |
| B02.1 | Zoster meningitis |
| B02.2 | Zoster with other nervous system involvement |
| B02.3 | Zoster ocular disease |
| B02.7 | Disseminated zoster |
| B02.8 | Zoster with other complications |
| B02.9 | Zoster without complication |
| B04* | Monkeypox |
| B05 | Measles |
| B05.0 | Measles complicated by encephalitis |
| B05.1 | Measles complicated by meningitis |
| B05.2 | Measles complicated by pneumonia |
| B05.3 | Measles complicated by otitis media |
| B05.4 | Measles with intestinal complications |
| B05.8 | Measles with other complications |
| B05.9 | Measles without complication |
| B06 | Rubella [German measles] |
| B06.0 | Rubella with neurological complications |
| B06.8 | Rubella with other complications |
| B06.9 | Rubella without complication |
| B07* | Viral warts |
| B08.0 | Other orthopoxvirus infections |
| B08.1 | Molluscum contagiosum |
| B08.2 | Exanthema subitum [sixth disease] |
| B08.3 | Erythema infectiosum [fifth disease] |
| B08.4 | Enteroviral vesicular stomatitis with exanthem |
| B08.5 | Enteroviral vesicular pharyngitis |
| B08.8 | Other specified viral infections characterized by skin and mucous membrane lesions |
| B09* | Unspecified viral infection characterized by skin and mucous membrane lesions |
| B15 | Acute hepatitis A |
| B15.0 | Hepatitis A with hepatic coma |
| B15.9 | Hepatitis A without hepatic coma |
| B16 | Acute hepatitis B |
| B16.0 | Acute hepatitis B with delta-agent (coinfection) with hepatic coma |
| B16.1 | Acute hepatitis B with delta-agent (coinfection) without hepatic coma |
| B16.2 | Acute hepatitis B without delta-agent with hepatic coma |
| B16.9 | Acute hepatitis B without delta-agent and without hepatic coma |
| B17* | Other acute viral hepatitis |
| B18* | Chronic viral hepatitis |
| B19 | Unspecified viral hepatitis |
| B19.0 | Unspecified viral hepatitis with hepatic coma |
| B19.9 | Unspecified viral hepatitis without hepatic coma |
| B20* | Human immunodeficiency virus [HIV] disease resulting in infectious and parasitic diseases |
| B21* | Human immunodeficiency virus [HIV] disease resulting in malignant neoplasms |
| B21.0 | HIV disease resulting in Kaposi sarcoma |
| B22 | Human immunodeficiency virus [HIV] disease resulting in other specified diseases |
| B22.0 | HIV disease resulting in encephalopathy |
| B22.1 | HIV disease resulting in lymphoid interstitial pneumonitis |
| B22.2 | HIV disease resulting in wasting syndrome |
| B22.7 | HIV disease resulting in multiple diseases classified elsewhere |
| B23* | Human immunodeficiency virus [HIV] disease resulting in other conditions |
| B24* | Unspecified human immunodeficiency virus [HIV] disease |
| B25* | Cytomegaloviral disease |
| B26 | Mumps |
| B26.1 | Mumps meningitis |
| B26.2 | Mumps encephalitis |
| B26.3 | Mumps pancreatitis |
| B26.8 | Mumps with other complications |
| B26.9 | Mumps without complication |
| B27 | Infectious mononucleosis |
| B27.0 | Gammaherpesviral mononucleosis |
| B27.1 | Cytomegaloviral mononucleosis |
| B27.8 | Other infectious mononucleosis |
| B27.9 | Infectious mononucleosis, unspecified |
| B30* | Viral conjunctivitis |
| B33 | Other viral diseases, not elsewhere classified |
| B33.0 | Epidemic myalgia |
| B33.1 | Ross River disease |
| B33.2 | Viral carditis |
| B33.3 | Retrovirus infections, not elsewhere classified |
| B33.4 | Hantavirus (cardio-)pulmonary syndrome [HPS] [HCPS] |
| B33.8 | Other specified viral diseases |
| B34 | Viral infection of unspecified site |
| B34.0 | Adenovirus infection, unspecified site |
| B34.1 | Enterovirus infection, unspecified site |
| B34.2 | Coronavirus infection, unspecified site |
| B34.3 | Parvovirus infection, unspecified site |
| B34.4 | Papovavirus infection, unspecified site |
| B34.8 | Other viral infections of unspecified site |
| B34.9 | Viral infection, unspecified |
| B35* | Dermatophytosis |
| B36* | Other superficial mycoses |
| B37 | Candidiasis |
| B37.0 | Candidal stomatitis |
| B37.1 | Pulmonary candidiasis |
| B37.2 | Candidiasis of skin and nail |
| B37.4 | Candidiasis of other urogenital sites |
| B37.5 | Candidal meningitis |
| B37.6 | Candidal endocarditis |
| B37.7 | Candidal sepsis |
| B37.8 | Candidiasis of other sites |
| B37.9 | Candidiasis, unspecified |
| B38 | Coccidioidomycosis |
| B38.0 | Acute pulmonary coccidioidomycosis |
| B38.1 | Chronic pulmonary coccidioidomycosis |
| B38.2 | Pulmonary coccidioidomycosis, unspecified |
| B38.3 | Cutaneous coccidioidomycosis |
| B38.4 | Coccidioidomycosis meningitis |
| B38.7 | Disseminated coccidioidomycosis |
| B38.8 | Other forms of coccidioidomycosis |
| B38.9 | Coccidioidomycosis, unspecified |
| B39 | Histoplasmosis |
| B39.0 | Acute pulmonary histoplasmosis capsulati |
| B39.1 | Chronic pulmonary histoplasmosis capsulati |
| B39.2 | Pulmonary histoplasmosis capsulati, unspecified |
| B39.3 | Disseminated histoplasmosis capsulati |
| B39.4 | Histoplasmosis capsulati, unspecified |
| B39.5 | Histoplasmosis duboisii |
| B39.9 | Histoplasmosis, unspecified |
| B40 | Blastomycosis |
| B40.0 | Acute pulmonary blastomycosis |
| B40.1 | Chronic pulmonary blastomycosis |
| B40.2 | Pulmonary blastomycosis, unspecified |
| B40.3 | Cutaneous blastomycosis |
| B40.7 | Disseminated blastomycosis |
| B40.8 | Other forms of blastomycosis |
| B40.9 | Blastomycosis, unspecified |
| B41 | Paracoccidioidomycosis |
| B41.0 | Pulmonary paracoccidioidomycosis |
| B41.7 | Disseminated paracoccidioidomycosis |
| B41.8 | Other forms of paracoccidioidomycosis |
| B41.9 | Paracoccidioidomycosis, unspecified |
| B42 | Sporotrichosis |
| B42.0 | Pulmonary sporotrichosis |
| B42.1 | Lymphocutaneous sporotrichosis |
| B42.7 | Disseminated sporotrichosis |
| B42.8 | Other forms of sporotrichosis |
| B42.9 | Sporotrichosis, unspecified |
| B43 | Chromomycosis and phaeomycotic abscess |
| B43.0 | Cutaneous chromomycosis |
| B43.1 | Phaeomycotic brain abscess |
| B43.2 | Subcutaneous phaeomycotic abscess and cyst |
| B43.8 | Other forms of chromomycosis |
| B43.9 | Chromomycosis, unspecified |
| B44* | Aspergillosis |
| B45 | Cryptococcosis |
| B45.0 | Pulmonary cryptococcosis |
| B45.1 | Cerebral cryptococcosis |
| B45.2 | Cutaneous cryptococcosis |
| B45.3 | Osseous cryptococcosis |
| B45.7 | Disseminated cryptococcosis |
| B45.8 | Other forms of cryptococcosis |
| B45.9 | Cryptococcosis, unspecified |
| B46 | Zygomycosis |
| B46.0 | Pulmonary mucormycosis |
| B46.1 | Rhinocerebral mucormycosis |
| B46.2 | Gastrointestinal mucormycosis |
| B46.3 | Cutaneous mucormycosis |
| B46.4 | Disseminated mucormycosis |
| B46.5 | Mucormycosis, unspecified |
| B46.8 | Other zygomycoses |
| B46.9 | Zygomycosis, unspecified |
| B47 | Mycetoma |
| B47.0 | Eumycetoma |
| B47.1 | Actinomycetoma |
| B47.9 | Mycetoma, unspecified |
| B48 | Other mycoses, not elsewhere classified |
| B48.0 | Lobomycosis |
| B48.1 | Rhinosporidiosis |
| B48.2 | Allescheriasis |
| B48.3 | Geotrichosis |
| B48.4 | Penicillosis |
| B48.7 | Opportunistic mycoses |
| B48.8 | Other specified mycoses |
| B49* | Unspecified mycosis |
| B50* | Plasmodium falciparum malaria |
| B50.0 | Plasmodium falciparum malaria with cerebral complications |
| B50.8 | Other severe and complicated Plasmodium falciparum malaria |
| B50.9 | Plasmodium falciparum malaria, unspecified |
| B51* | Plasmodium vivax malaria |
| B52* | Plasmodium malariae malaria |
| B53 | Other parasitologically confirmed malaria |
| B53.0 | Plasmodium ovale malaria |
| B53.1 | Malaria due to simian plasmodia |
| B53.8 | Other parasitologically confirmed malaria, not elsewhere classified |
| B54* | Unspecified malaria |
| B55.0 | Visceral leishmaniasis |
| B55.1 | Cutaneous leishmaniasis |
| B55.2 | Mucocutaneous leishmaniasis |
| B55.9 | Leishmaniasis, unspecified |
| B56* | African trypanosomiasis |
| B57* | Chagas disease |
| B58 | Toxoplasmosis |
| B58.0 | Toxoplasma oculopathy |
| B58.1 | Toxoplasma hepatitis |
| B58.2 | Toxoplasma meningoencephalitis |
| B58.3 | Pulmonary toxoplasmosis |
| B58.8 | Toxoplasmosis with other organ involvement |
| B58.9 | Toxoplasmosis, unspecified |
| B59* | Pneumocystosis |
| B60.0 | Babesiosis |
| B60.1 | Acanthamoebiasis |
| B60.2 | Naegleriasis |
| B60.8 | Other specified protozoal diseases |
| B64* | Unspecified protozoal disease |
| B65 | Schistosomiasis [bilharziasis] |
| B65.0 | Schistosomiasis due to Schistosoma haematobium [urinary schistosomiasis] |
| B65.1 | Schistosomiasis due to Schistosoma mansoni [intestinal schistosomiasis] |
| B65.2 | Schistosomiasis due to Schistosoma japonicum |
| B65.3 | Cercarial dermatitis |
| B65.8 | Other schistosomiases |
| B65.9 | Schistosomiasis, unspecified |
| B66 | Other fluke infections |
| B66.0 | Opisthorchiasis |
| B66.1 | Clonorchiasis |
| B66.2 | Dicrocoeliasis |
| B66.3 | Fascioliasis |
| B66.4 | Paragonimiasis |
| B66.5 | Fasciolopsiasis |
| B66.8 | Other specified fluke infections |
| B66.9 | Fluke infection, unspecified |
| B67* | Echinococcosis |
| B68 | Taeniasis |
| B68.0 | Taenia solium taeniasis |
| B68.1 | Taenia saginata taeniasis |
| B68.9 | Taeniasis, unspecified |
| B69 | Cysticercosis |
| B69.0 | Cysticercosis of central nervous system |
| B69.1 | Cysticercosis of eye |
| B69.8 | Cysticercosis of other sites |
| B69.9 | Cysticercosis, unspecified |
| B70 | Diphyllobothriasis and sparganosis |
| B70.0 | Diphyllobothriasis |
| B70.1 | Sparganosis |
| B71 | Other cestode infections |
| B71.0 | Hymenolepiasis |
| B71.1 | Dipylidiasis |
| B71.8 | Other specified cestode infections |
| B71.9 | Cestode infection, unspecified |
| B72* | Dracunculiasis |
| B73* | Onchocerciasis |
| B74* | Filariasis |
| B75* | Trichinellosis |
| B76* | Hookworm diseases |
| B77* | Ascariasis |
| B78* | Strongyloidiasis |
| B79* | Trichuriasis |
| B80* | Enterobiasis |
| B81 | Other intestinal helminthiases, not elsewhere classified |
| B81.0 | Anisakiasis |
| B81.1 | Intestinal capillariasis |
| B81.2 | Trichostrongyliasis |
| B81.3 | Intestinal angiostrongyliasis |
| B81.4 | Mixed intestinal helminthiases |
| B81.8 | Other specified intestinal helminthiases |
| B82 | Unspecified intestinal parasitism |
| B82.0 | Intestinal helminthiasis, unspecified |
| B82.9 | Intestinal parasitism, unspecified |
| B83 | Other helminthiases |
| B83.0 | Visceral larva migrans |
| B83.1 | Gnathostomiasis |
| B83.2 | Angiostrongyliasis due to Parastrongylus cantonensis |
| B83.3 | Syngamiasis |
| B83.4 | Internal hirudiniasis |
| B83.8 | Other specified helminthiases |
| B83.9 | Helminthiasis, unspecified |
| B85* | Pediculosis and phthiriasis |
| B86* | Scabies |
| B87* | Myiasis |
| B88 | Other infestations |
| B88.0 | Other acariasis |
| B88.1 | Tungiasis [sandflea infestation] |
| B88.2 | Other arthropod infestations |
| B88.3 | External hirudiniasis |
| B88.8 | Other specified infestations |
| B88.9 | Infestation, unspecified |
| B89* | Unspecified parasitic disease |
| B95* | Streptococcus and staphylococcus as the cause of diseases classified to other chapters |
| B96.0 | Mycoplasma pneumoniae [M. pneumoniae] as the cause of diseases classified to other chapters |
| B96.1 | Klebsiella pneumoniae [K. pneumoniae] as the cause of diseases classified to other chapters |
| B96.2 | Escherichia coli [E. coli] as the cause of diseases classified to other chapters |
| B96.3 | Haemophilus influenzae [H. influenzae] as the cause of diseases classified to other chapters |
| B96.4 | Proteus (mirabilis)(morganii) as the cause of diseases classified to other chapters |
| B96.5 | Pseudomonas (aeruginosa) as the cause of diseases classified to other chapters |
| B96.6 | Bacillus fragilis [B. fragilis] as the cause of diseases classified to other chapters |
| B96.7 | Clostridium perfringens [C. perfringens] as the cause of diseases classified to other chapters |
| B96.8 | Other specified bacterial agents as the cause of diseases classified to other chapters |
| B97 | Viral agents as the cause of diseases classified to other chapters |
| B97.0 | Adenovirus as the cause of diseases classified to other chapters |
| B97.1 | Enterovirus as the cause of diseases classified to other chapters |
| B97.2 | Coronavirus as the cause of diseases classified to other chapters |
| B97.3 | Retrovirus as the cause of diseases classified to other chapters |
| B97.4 | Respiratory syncytial virus as the cause of diseases classified to other chapters |
| B97.5 | Reovirus as the cause of diseases classified to other chapters |
| B97.6 | Parvovirus as the cause of diseases classified to other chapters |
| B97.7 | Papillomavirus as the cause of diseases classified to other chapters |
| B97.8 | Other viral agents as the cause of diseases classified to other chapters |
| B98.0 | Helicobacter pylori [H.pylori] as the cause of diseases classified to other chapters |
| B98.1 | Vibrio vulnificus as the cause of diseases classified to other chapters |
| B99* | Other and unspecified infectious diseases |
| C46 | Kaposi sarcoma |
| D73.3 | Abscess of spleen |
| E32.1 | Abscess of thymus |
| G00 | Bacterial meningitis, not elsewhere classified |
| G00.0 | Haemophilus meningitis |
| G00.1 | Pneumococcal meningitis |
| G00.2 | Streptococcal meningitis |
| G00.3 | Staphylococcal meningitis |
| G00.8 | Other bacterial meningitis |
| G00.9 | Bacterial meningitis, unspecified |
| G01* | Meningitis in bacterial diseases classified elsewhere |
| G02.0 | Meningitis in viral diseases classified elsewhere |
| G02.1 | Meningitis in mycoses |
| G02.8 | Meningitis in other specified infectious and parasitic diseases classified elsewhere |
| G03* | Meningitis due to other and unspecified causes |
| G04.1 | Tropical spastic paraplegia |
| G04.2 | Bacterial meningoencephalitis and meningomyelitis, not elsewhere classified |
| G05.0 | Encephalitis, myelitis and encephalomyelitis in bacterial diseases classified elsewhere |
| G05.1 | Encephalitis, myelitis and encephalomyelitis in viral diseases classified elsewhere |
| G05.2 | Encephalitis, myelitis and encephalomyelitis in other infectious and parasitic diseases classified elsewhere |
| G06* | Intracranial and intraspinal abscess and granuloma |
| G07* | Intracranial and intraspinal abscess and granuloma in diseases classified elsewhere |
| H00* | Hordeolum and chalazion |
| H01.0 | Blepharitis |
| H05.0 | Acute inflammation of orbit |
| H06.1 | Parasitic infestation of orbit in diseases classified elsewhere |
| H10.0 | Mucopurulent conjunctivitis |
| H10.5 | Blepharoconjunctivitis |
| H13.0 | Filarial infection of conjunctiva |
| H19.0 | Scleritis and episcleritis in diseases classified elsewhere |
| H19.1 | Herpesviral keratitis and keratoconjunctivitis |
| H19.2 | Keratitis and keratoconjunctivitis in other infectious and parasitic diseases classified elsewhere |
| H22.0 | Iridocyclitis in infectious and parasitic diseases classified elsewhere |
| H32.0 | Chorioretinal inflammation in infectious and parasitic diseases classified elsewhere |
| H44.0 | Purulent endophthalmitis |
| H60.0 | Abscess of external ear |
| H60.1 | Cellulitis of external ear |
| H60.2 | Malignant otitis externa |
| H60.3 | Other infective otitis externa |
| H62.0 | Otitis externa in bacterial diseases classified elsewhere |
| H62.1 | Otitis externa in viral diseases classified elsewhere |
| H62.2 | Otitis externa in mycoses |
| H62.3 | Otitis externa in other infectious and parasitic diseases classified elsewhere |
| H66.0 | Acute suppurative otitis media |
| H67.0 | Otitis media in bacterial diseases classified elsewhere |
| H67.1 | Otitis media in viral diseases classified elsewhere |
| H70.0 | Acute mastoiditis |
| H75.0 | Mastoiditis in infectious and parasitic diseases classified elsewhere |
| I30.1 | Infective pericarditis |
| I32.0 | Pericarditis in bacterial diseases classified elsewhere |
| I32.1 | Pericarditis in other infectious and parasitic diseases classified elsewhere |
| I33.0 | Acute and subacute infective endocarditis |
| I40.0 | Infective myocarditis |
| I41.0 | Myocarditis in bacterial diseases classified elsewhere |
| I41.1 | Myocarditis in viral diseases classified elsewhere |
| I41.2 | Myocarditis in other infectious and parasitic diseases classified elsewhere |
| I43.0 | Cardiomyopathy in infectious and parasitic diseases classified elsewhere |
| I52.0 | Other heart disorders in bacterial diseases classified elsewhere |
| I52.1 | Other heart disorders in other infectious and parasitic diseases classified elsewhere |
| I68.1 | Cerebral arteritis in infectious and parasitic diseases classified elsewhere |
| J01.0 | Acute maxillary sinusitis |
| J02 | Acute pharyngitis |
| J02.0 | Streptococcal pharyngitis |
| J02.8 | Acute pharyngitis due to other specified organisms |
| J02.9 | Acute pharyngitis, unspecified |
| J03 | Acute tonsillitis |
| J03.0 | Streptococcal tonsillitis |
| J03.8 | Acute tonsillitis due to other specified organisms |
| J03.9 | Acute tonsillitis, unspecified |
| J04* | Acute laryngitis and tracheitis |
| J05.1 | Acute epiglottitis |
| J09* | Influenza due to certain identified influenza virus |
| J10* | Influenza due to other identified influenza virus |
| J11* | Influenza, virus not identified |
| J12* | Viral pneumonia, not elsewhere classified |
| J13* | Pneumonia due to Streptococcus pneumoniae |
| J14* | Pneumonia due to Haemophilus influenzae |
| J15 | Bacterial pneumonia, not elsewhere classified |
| J15.0 | Pneumonia due to Klebsiella pneumoniae |
| J15.1 | Pneumonia due to Pseudomonas |
| J15.2 | Pneumonia due to staphylococcus |
| J15.3 | Pneumonia due to streptococcus, group B |
| J15.4 | Pneumonia due to other streptococci |
| J15.5 | Pneumonia due to Escherichia coli |
| J15.6 | Pneumonia due to other aerobic Gram-negative bacteria |
| J15.7 | Pneumonia due to Mycoplasma pneumoniae |
| J15.8 | Other bacterial pneumonia |
| J15.9 | Bacterial pneumonia, unspecified |
| J16 | Pneumonia due to other infectious organisms, not elsewhere classified |
| J16.0 | Chlamydial pneumonia |
| J16.8 | Pneumonia due to other specified infectious organisms |
| J17.0 | Pneumonia in bacterial diseases classified elsewhere |
| J17.1 | Pneumonia in viral diseases classified elsewhere |
| J17.2 | Pneumonia in mycoses |
| J17.3 | Pneumonia in parasitic diseases |
| J17.8 | Pneumonia in other diseases classified elsewhere |
| J18* | Pneumonia, organism unspecified |
| J20 | Acute bronchitis |
| J20.0 | Acute bronchitis due to Mycoplasma pneumoniae |
| J20.1 | Acute bronchitis due to Haemophilus influenzae |
| J20.2 | Acute bronchitis due to streptococcus |
| J20.3 | Acute bronchitis due to coxsackievirus |
| J20.4 | Acute bronchitis due to parainfluenza virus |
| J20.5 | Acute bronchitis due to respiratory syncytial virus |
| J20.6 | Acute bronchitis due to rhinovirus |
| J20.7 | Acute bronchitis due to echovirus |
| J20.8 | Acute bronchitis due to other specified organisms |
| J20.9 | Acute bronchitis, unspecified |
| J21* | Acute bronchiolitis |
| J21.0 | Acute bronchiolitis due to respiratory syncytial virus |
| J21.1 | Acute bronchiolitis due to human metapneumovirus |
| J21.8 | Acute bronchiolitis due to other specified organisms |
| J21.9 | Acute bronchiolitis, unspecified |
| J22* | Unspecified acute lower respiratory infection |
| J36* | Peritonsillar abscess |
| J39.0 | Retropharyngeal and parapharyngeal abscess |
| J39.1 | Other abscess of pharynx |
| J85.1 | Abscess of lung with pneumonia |
| J85.2 | Abscess of lung without pneumonia |
| J85.3 | Abscess of mediastinum |
| J86* | Pyothorax |
| K02* | Dental caries |
| K04.4 | Acute apical periodontitis of pulpal origin |
| K04.5 | Chronic apical periodontitis |
| K04.6 | Periapical abscess with sinus |
| K04.7 | Periapical abscess without sinus |
| K05.0 | Acute gingivitis |
| K05.2 | Acute periodontitis |
| K05.3 | Chronic periodontitis |
| K05.4 | Periodontosis |
| K11.3 | Abscess of salivary gland |
| K12.2 | Cellulitis and abscess of mouth |
| K23.0 | Tuberculous oesophagitis |
| K23.1 | Megaoesophagus in Chagas disease |
| K35* | Acute appendicitis |
| K57.0 | Diverticular disease of small intestine with perforation and abscess |
| K57.2 | Diverticular disease of large intestine with perforation and abscess |
| K57.4 | Diverticular disease of both small and large intestine with perforation and abscess |
| K57.8 | Diverticular disease of intestine, part unspecified, with perforation and abscess |
| K61* | Abscess of anal and rectal regions |
| K63.0 | Abscess of intestine |
| K65.0 | Acute peritonitis |
| K67.0 | Chlamydial peritonitis |
| K67.1 | Gonococcal peritonitis |
| K67.2 | Syphilitic peritonitis |
| K67.3 | Tuberculous peritonitis |
| K67.8 | Other disorders of peritoneum in infectious diseases classified elsewhere |
| K75.0 | Abscess of liver |
| K77.0 | Liver disorders in infectious and parasitic diseases classified elsewhere |
| L00* | Staphylococcal scalded skin syndrome |
| L01* | Impetigo |
| L02* | Cutaneous abscess, furuncle and carbuncle |
| L03* | Cellulitis |
| L04* | Acute lymphadenitis |
| L05* | Pilonidal cyst |
| L08* | Other local infections of skin and subcutaneous tissue |
| L70.1 | Acne conglobata |
| M00 | Pyogenic arthritis |
| M00.0 | Staphylococcal arthritis and polyarthritis |
| M00.1 | Pneumococcal arthritis and polyarthritis |
| M00.2 | Other streptococcal arthritis and polyarthritis |
| M00.8 | Arthritis and polyarthritis due to other specified bacterial agents |
| M00.9 | Pyogenic arthritis, unspecified |
| M01.0 | Meningococcal arthritis |
| M01.1 | Tuberculous arthritis |
| M01.2 | Arthritis in Lyme disease |
| M01.3 | Arthritis in other bacterial diseases classified elsewhere |
| M01.4 | Rubella arthritis |
| M01.5 | Arthritis in other viral diseases classified elsewhere |
| M01.6 | Arthritis in mycoses |
| M01.8 | Arthritis in other infectious and parasitic diseases classified elsewhere |
| M46.2 | Osteomyelitis of vertebra |
| M46.3 | Infection of intervertebral disc (pyogenic) |
| M46.5 | Other infective spondylopathies |
| M49.0 | Tuberculosis of spine |
| M49.1 | Brucella spondylitis |
| M49.2 | Enterobacterial spondylitis |
| M49.3 | Spondylopathy in other infectious and parasitic diseases classified elsewhere |
| M60.0 | Infective myositis |
| M63.0 | Myositis in bacterial diseases classified elsewhere |
| M63.1 | Myositis in protozoal and parasitic infections classified elsewhere |
| M63.2 | Myositis in other infectious diseases classified elsewhere |
| M65.0 | Abscess of tendon sheath |
| M65.1 | Other infective (teno)synovitis |
| M71.0 | Abscess of bursa |
| M71.1 | Other infective bursitis |
| M72.6 | Necrotizing fasciitis |
| M73.0 | Gonococcal bursitis |
| M73.1 | Syphilitic bursitis |
| M86* | Osteomyelitis |
| M86.0 | Acute haematogenous osteomyelitis |
| M86.1 | Other acute osteomyelitis |
| M86.2 | Subacute osteomyelitis |
| M86.3 | Chronic multifocal osteomyelitis |
| M86.4 | Chronic osteomyelitis with draining sinus |
| M86.5 | Other chronic haematogenous osteomyelitis |
| M86.6 | Other chronic osteomyelitis |
| M86.8 | Other osteomyelitis |
| M86.9 | Osteomyelitis, unspecified |
| N08.0 | Glomerular disorders in infectious and parasitic diseases classified elsewhere |
| N10* | Acute tubulo-interstitial nephritis |
| N13.6 | Pyonephrosis |
| N15.1 | Renal and perinephric abscess |
| N16.0 | Renal tubulo-interstitial disorders in infectious and parasitic diseases classified elsewhere |
| N29.0 | Late syphilis of kidney |
| N29.1 | Other disorders of kidney and ureter in infectious and parasitic diseases classified elsewhere |
| N30.0 | Acute cystitis |
| N34.0 | Urethral abscess |
| N39.0 | Urinary tract infection, site not specified |
| N61* | Inflammatory disorders of breast |
| P23 | Congenital pneumonia |
| P23.0 | Congenital pneumonia due to viral agent |
| P23.1 | Congenital pneumonia due to Chlamydia |
| P23.2 | Congenital pneumonia due to staphylococcus |
| P23.3 | Congenital pneumonia due to streptococcus, group B |
| P23.4 | Congenital pneumonia due to Escherichia coli |
| P23.5 | Congenital pneumonia due to Pseudomonas |
| P23.6 | Congenital pneumonia due to other bacterial agents |
| P23.8 | Congenital pneumonia due to other organisms |
| P23.9 | Congenital pneumonia, unspecified |
| P35.0 | Congenital rubella syndrome |
| P35.1 | Congenital cytomegalovirus infection |
| P35.2 | Congenital herpesviral [herpes simplex] infection |
| P35.3 | Congenital viral hepatitis |
| P36 | Bacterial sepsis of newborn |
| P36.0 | Sepsis of newborn due to streptococcus, group B |
| P36.1 | Sepsis of newborn due to other and unspecified streptococci |
| P36.2 | Sepsis of newborn due to Staphylococcus aureus |
| P36.3 | Sepsis of newborn due to other and unspecified staphylococci |
| P36.4 | Sepsis of newborn due to Escherichia coli |
| P36.5 | Sepsis of newborn due to anaerobes |
| P36.8 | Other bacterial sepsis of newborn |
| P36.9 | Bacterial sepsis of newborn, unspecified |
| P37 | Other congenital infectious and parasitic diseases |
| P37.0 | Congenital tuberculosis |
| P37.1 | Congenital toxoplasmosis |
| P37.2 | Neonatal (disseminated) listeriosis |
| P37.3 | Congenital falciparum malaria |
| P37.4 | Other congenital malaria |
| P37.5 | Neonatal candidiasis |
| P37.8 | Other specified congenital infectious and parasitic diseases |
| P37.9 | Congenital infectious and parasitic disease, unspecified |
| P38* | Omphalitis of newborn with or without mild haemorrhage |
| P39.0 | Neonatal infective mastitis |
| P39.1 | Neonatal conjunctivitis and dacryocystitis |
| P39.2 | Intra-amniotic infection of fetus, not elsewhere classified |
| P39.3 | Neonatal urinary tract infection |
| P39.4 | Neonatal skin infection |
| R57.2 | Septic shock |
| R65.0 | Systemic Inflammatory Response Syndrome of infectious origin without organ failure |
| R65.1 | Systemic Inflammatory Response Syndrome of infectious origin with organ failure |
| Z21 | Asymptomatic human immunodeficiency virus [HIV] infection status |

The list above comprises almost all codes under Chapter I (Certain infectious and parasitic diseases) of ICD-10 and additionally infectious disease codes from other Chapters of ICD-10. The few excluded codes include sequelae of infectious and parasitic diseases when the infection is not anymore present [B90-B94], prion diseases and other very rare central nervous system conditions [A81.0, A81.8, A81.9], rabies [A82], smallbox [B03], common cold [J00], acute upper respiratory infections of multiple and unspecified sites [J06], complications of surgical and medical care, not elsewhere classified [T80-T88], codes for special purposes [U00-U85], and sex-specific infections. Notably, consistent with previous large-scale studies, ^1^ specific emergency codes for COVID-19 were excluded from our primary outcome definition to avoid potential confounding from pandemic-specific hospitalization policies, although general coronavirus infections were included.

* Indicates inclusion of underlying 4- and 5-digit codes.

**References**

1 Elovainio M, Komulainen K, Sipilä PN, *et al.* Association of social isolation and loneliness with risk of incident hospital-treated infections: an analysis of data from the UK Biobank and Finnish Health and Social Support studies. *Lancet Public Health* 2023; **8**: e109–18.

**Appendix S2. Classification of site-specific hospital-treated infectious diseases in the study on diagnosis level**

| **Subtype of infectious diseases** | **ICD codes** |
| --- | --- |
| Upper respiratory tract, including ear | A54.5, B05.3, B08.5, B97.4, H60.0, H60.1, H60.2, H60.3, H62.0, H62.1, H62.2, H62.3, H66.0, H67.0, H67.1, H70.0, H75.0, J01.0, J02, J02.0, J02.8, J02.9, J03, J03.0, J03.8, J03.9, J04*, J05.1, J36*, J39.0, J39.1 |
| Lower respiratory tract | A06.5, A15*, A16*, A20.2, A21.0, A21.2, A22.1, A42.0, A43.0, B01.2, B05.2, B22.1, B37.1, B38.0, B38.1, B38.2, B40.0, B40.1, B40.2, B41.0, B42.0, B45.0, B46.0, B58.3, B59*, B66.4, B96.0, B96.1, J12*, J13*, J14*, J15, J15.0, J15.1, J15.2, J15.3, J15.4, J15.5, J15.6, J15.7, J15.8, J15.9, J16, J16.0, J16.8, J17.0, J17.1, J17.2, J17.3, J17.8, J18*, J20, J20.0, J20.1, J20.2, J20.3, J20.4, J20.5, J20.6, J20.7, J20.8, J20.9, J21*, J21.0, J21.1, J21.8, J21.9, J22*, J85.1, J85.2, J85.3, J86*, P23, P23.0, P23.1, P23.2, P23.3, P23.4, P23.5, P23.6, P23.8, P23.9, P37.0 |
| Gastrointestinal tract, including liver | A00, A00.0, A00.1, A00.9, A01*, A02, A02.0, A02.2, A02.8, A02.9, A03, A03.0, A03.1, A03.2, A03.3, A03.8, A03.9, A04, A04.0, A04.1, A04.2, A04.3, A04.4, A04.5, A04.6, A04.7, A04.8, A04.9, A05, A05.0, A05.1, A05.2, A05.3, A05.4, A05.8, A05.9, A06, A06.0, A06.1, A06.2, A06.3, A06.4, A07*, A08.0, A08.1, A08.2, A08.3, A08.4, A08.5, A09*, A22.2, B05.4, B15, B15.0, B15.9, B16, B16.0, B16.1, B16.2, B16.9, B17*, B18*, B19, B19.0, B19.9, B46.2, B57*, B58.1, B65, B66.3, B81, B81.0, B81.1, B81.2, B81.3, B81.4, B81.8, B97.1, B98.0, K35*, K57.0, K57.2, K57.4, K57.8, K61*, K63.0, K65.0, K67.0, K67.1, K67.2, K67.3, K67.8, K75.0, K77.0, P35.3 |
| Genitourinary | A51.0, A51.1, A54.0, A54.1, A54.2, A54.6, A54.8, A54.9, A55*, A56.0, A56.1, A56.2, A56.3, A56.4, A56.8, A57*, A58*, A59*, A60*, A63.0, A63.8, A64*, B37.4, N08.0, N10*, N13.6, N15.1, N16.0, N29.0, N29.1, N30.0, N34.0, N39.0, N61*, P39.0, P39.3 |
| Bloodstream | A02.1, A20.7, A21.7, A21.8, A21.9, A22.7, A26.7, A32.7, A39.1, A39.2, A39.3, A39.4, A40, A40.0, A40.1, A40.2, A40.3, A40.8, A40.9, A41, A41.0, A41.1, A41.2, A41.3, A41.4, A41.5, A41.8, A41.9, A42.7, A44.0, A48.3, A48.4, B37.7, B65.0, B65.1, B65.2, B65.3, B65.8, B65.9, P36, P36.0, P36.1, P36.2, P36.3, P36.4, P36.5, P36.8, P36.9, P37.2, R57.2, R65.0, R65.1 |
| Heart and circulation | A52.0, B33.2, B33.4, B37.6, I30.1, I32.0, I32.1, I33.0, I40.0, I41.0, I41.1, I41.2, I43.0, I52.0, I52.1, I68.1, |
| Bone, joint, and connective tissue | A54.4, B33.0, B45.3, M00, M00.0, M00.1, M00.2, M00.8, M00.9, M01.0, M01.1, M01.2, M01.3, M01.4, M01.5, M01.6, M01.8, M46.2, M46.3, M46.5, M49.0, M49.1, M49.2, M49.3, M60.0, M63.0, M63.1, M63.2, M65.0, M65.1, M71.0, M71.1, M72.6, M73.0, M73.1, M86*, M86.0, M86.1, M86.2, M86.3, M86.4, M86.5, M86.6, M86.8, M86.9 |
| Neurological and eye | A06.6, A17, A17.0, A17.1, A17.8, A17.9, A20.3, A21.1, A32.1, A39, A39.0, A39.5, A39.8, A39.9, A50.3, A50.4, A52.1, A52.2, A52.3, A54.3, A71*, A74.0, A80*, A81.1, A81.2, A83*, A84*, A85*, A86*, A87*, A88*, A89*, B00.3, B00.4, B00.5, B01.0, B01.1, B02.0, B02.1, B02.2, B02.3, B05.0, B05.1, B06.0, B26.1, B26.2, B30*, B37.5, B38.4, B45.1, B46.1, B58.0, B58.2, B69.0, B69.1, G00, G00.0, G00.1, G00.2, G00.3, G00.8, G00.9, G01*, G02.0, G02.1, G02.8, G03*, G04.1, G04.2, G05.0, G05.1, G05.2, G06*, G07*, H00*, H01.0, H05.0, H06.1, H10.0, H10.5, H13.0, H19.0, H19.1, H19.2, H22.0, H32.0, H44.0, P39.1 |
| Skin and soft tissue | A06.7, A20.1, A22.0, A26.0, A32.0, A43.1, A44.1, A51.3, A67*, B00.0, B00.1, B07*, B08.8, B09*, B35*, B37.2, B38.3, B43, B43.0, B43.1, B43.2, B43.8, B45.2, B46.3, B48.0, B55.1, B55.2, B86*, L00*, L01*, L02*, L03*, L04*, L05*, L08*, L70.1, P39.4 |

**Appendix S3. The codes and classification of the participants’ jobs**

| **1. Healthcare and Nursing**  **·health (human or animal), residential/social/religious care, undertaking (including managers)**  Coding:  -3133064,-3133065,22113066,22123067,22133068,22143069,22153070,-3133071,  32113072,61113073,32123074,32133075,61133076,32113077,32213078,32223079,32233080,32153081, 32293082,-3133083,32143084,32183085,31113086,321630  87,32173088,32183089,61123090,92213091,62913092,41313093,42113094,42163095,-3133096,11813097,11823098,11833099,11853100,11853101,-3133102,61143  103,61153104,61223105,32113106,-3133107,24423108,24433109,32313110, 3232  3111,32323112,32323113,24443114,11843115,-3133116,12393117,62913118,629  13119,62323120,91293121,53123122,-3133123,22163124,61313125,61393126,51  193127,51193128,51193129,91193130,91193131,34133132,51193133,12193134 |
| --- |
| **2. Agriculture, Natural Resources, and Environmental Engineering**  **·agriculture, horticulture, fishing, other work with animals (including managers)**  **·mining, quarrying, energy production, water treatment (including managers)**  **·cleaning, caretaking, waste collection, pest control (including managers)**  Coding:  -3132474,-3132475,51112476,12112477,91112478,82232479,91192480,91192481,  22162482,61312483,-3132484,12112485,51122486,91192487,51132488,82232489  ,51192490,91122491,12192492,12122493,35512494,35522495,-3132496,1219249  7,12192498,51192499,91192500,51192501,91192502,91192503,91192504,221625  05,61312506,-3132507,22162508,61312509,12192510,51192511,51192512,91192  513,51192514,12192515,34132516,61392517,12192518,51192519,12342520,51192521,-3132846,-3132847,81222848,82212849,81342850,81232851,82292852,8124  2853,81262854,91322855,52232856,52162857,81292858,91392859,-3132860,112  32861,11232862,24342863,21212864,21222865,21232866,31122867,31132868,21  252869,21282870,21292871,35662872,81332873,35672874,-3132966,-3132967,9  1322968,91322969,91322970,91322971,91322972,91322973,91322974,91322975,91322976,91322977,91322978,91322979,91322980,91322981,91322982,91322983,-3132984,92332985,92332986,92392987,92312988,92322989,92322990,923329  1,92342992,12392993,12392994,-3132995,62312996,62322997,62322998,922229  99,62323000,92223001,92213002,91493003,91493004,91493005,92493006,-3133  007,92353008,12353009,12353010,81293011,82113012,-3133013,62923014,6292  3015,12393016 |
| **3. Public, Business, and Service Sectors**  **·selling and shop work (retail/wholesale), storage and distribution (including managers)**  **·personal services, travel/tourism, hospitality (including managers)**  **·education, school-related work (including managers)**  **·sport, culture, arts, media, entertainment (including managers)**  **·armed forces, emergency services, security, health & safety (including managers)**  Coding:  -3133135,-3133136,34413137,34423138,34423139,12253140,34433141,62113142,  34493143,92293144,41233145,-3133146,34113147,34113148,34113149,34213150  ,34223151,34223152,34223153,34223154,34223155,-3133156,34123157,3412315  8,34313159,34323160,34343161,34333162,11343163,23293164,-3133165,341331  66,34133167,34133168,34143169,34153170,34133171,-3133172,12253173,12223  174,35393175,34163176,12253177,12243178,12393179,-3133180,62113181,6222  3182,92293183,41233184,92293185,34343186,92263187,24523188,62113189,92493190,-3133191,92293192,35663193,62113194,62113195,92493196,92263197,-3133319,-3133320,12343321,11633322,12313323,11423324,72123325,11633326  ,12323327,71113328,71123329,71253330,92513331,71293332,92413333,92593334,71293335,-3133336,71213337,71223338,71233339,71243340,72113341,711333  42,71293343,71293344,41233345,92593346,-3133347,11613348,11623349,41333  350,41343351,92113352,91493353,91493354,91493355,-3133356,-3133357,1233  3358,62213359,62223360,32293361,62223362,62223363,62113364,62223365,62223366,-3133367,12263368,62123369,62133370,-3133371,62143372,62193373,62  153374,62153375,62153376,92233377,62193378,62193379,82133380,62133381,-3133382,12233383,62113384,54343385,54323386,92233387,71123388,92243389,12243390,92253391,12213392,92263393,92223394,92453395,92333396,-313339  7,-3133398,23113399,23123400,23143401,23153402,23163403,23193404,23193  405,**-**3133406,42133407,61233408,61243409,32323410,92443411,92233412,623  23413,92433414,92333415,61213416,61223417,24513418,-3133419,23133420,2  3133421,23173422,42133423,24513424,41353425,24523426,41313427,41363428,23193429,23193430,-3133431,-3133432,11713433,33113434,-3133435,32133436,  61123437,11733438,11733439,33133440,11723441,33123442,41313443,33193444,41423445,-3133446,11733447,33143448,24433449,61143450,11733451,331934  52,-3133453,11743454,92413455,92423456,92453457,92493458,92493459,-3133  460,31233461,35653462,35653463,35663464,35663465,35663466,35673467,35683468 |
| **4. Manufacturing, Construction, and Transportation**  **·construction, building, demolition or maintenance (including managers)**  **·transport (road, rail, air, water), work with other mobile machinery (including managers)**  **·routine factory-based manufacturing (including managers)**  **·skilled manual work (including managers)**  Coding:  -3132522,-3132523,53122524,53122525,53132526,52152527,52412528,53152529  ,52162530,81492531,82292532,82212533,53192534,81492535,91212536,81492537,-3132538,53142539,53142540,52152541,52162542,52412543,81492544,814925  45,53152546,53162547,81492548,53192549,91212550,-3132551,53212552,53222  553,53222554,81492555,53232556,53152557,53232558,53232559,81492560,53192561,91212562,-3132563,53112564,81412565,53192566,53232567,52152568,822  12569,91212570,-3132571,81422572,81422573,82292574,82212575,81432576,81  412577,81412578,53232579,52152580,52142581,53152582,-3132583,91292584,9  1292585,91292586,91292587,91292588,81422589,-3132590,11222591,21212592,  81422593,81332594,35672595,35632596,24312597,24332598,24342599,24342600,-3132601,-3132602,82112603,82112604,82122605,82132606,62192607,821926  08,62132609,92332610,82142611,82152612,92452613,92422614,81342615,-3132  616,82212617,82222618,82292619,82292620,82292621,82232622,-3132623,3514  2624,82162625,82162626,82162627,62152628,62152629,92332630,82132631,524  92632,82162633,52232634,-3132635,35122636,35122637,62142638,35112639,52  232640,52232641,82182642,82182643,92332644,33192645,-3132646,35132647,3  5132648,35132649,62192650,82172651,82292652,92332653,91412654,82172655,82192656,33192657,-3132658,12322659,52312660,52322661,52332662,5223266  3,52342664,52492665,53152666,52142667,-3132668,11612669,41342670,116126  71,41342672,11612673,41342674,11612675,41342676,-3132677,-3132678,21222  679,21232680,21242681,21252682,21262683,21272684,21282685,21292686,31122687,31132688,52152689,52162690,-3132691,11212692,11212693,11412694,311  52695,35652696,35672697,35632698,**-**3132699,81112700,91342701,81332702,81  382703,81342704,91492705,91322706,81392707,91392708,-3132709,81132710,8  1142711,81362712,81372713,91342714,81332715,81382716,81342717,91492718,91322719,81392720,91392721,-3132722,81142723,91342724,81332725,81382726  ,81342727,91492728,91322729,81392730,91392731,-3132732,81122733,9134273  4,81332735,81382736,81342737,91492738,91322739,81392740,91392741,-31327  42,81162743,91342744,81332745,81382746,81342747,91492748,91322749,81392750,91392751,-3132752,81152753,91342754,81332755,81382756,81342757,9149  2758,91322759,81392760,91392761,-3132762,81142763,81182764,91342765,813  32766,81382767,81342768,91492769,91322770,81392771,91392772,-3132773,81  212774,91342775,81332776,81382777,81342778,91492779,91322780,81392781,91392782,-3132783,81192784,91342785,81332786,81382787,81342788,91492789,  91322790,81392791,91392792,-3132793,81312794,81312795,91342796,81332797  ,81342798,91492799,91322800,81392801,91392802,-3132803,81322804,5234280  5,91342806,81332807,81352808,81342809,91492810,91322811,81392812,91392813,-3132814,81322815,91342816,81332817,81342818,91492819,91322820,81392  821,91392822,-3132823,81392824,91342825,81332826,81382827,81342828,9149  2829,91322830,81392831,91392832,-3132833,81172834,81252835,81182836,812  92837,81292838,81382839,81342840,91492841,91322842,81392843,91312844,91392845,-3132875,-3132876,54112877,54122878,54132879,54142880,54192881,54  192882,54192883,54192884,-3132885,54212886,54222887,54232888,54242889,9  1332890,-3132891,54312892,54322893,54332894,54332895,54342896,21292897,-3132898,54912899,54912900,53122901,54912902,-3132903,54922904,53232905,  54922906,54922907,54922908,54942909,54942910,54932911,54932912,-3132913  ,54952914,54952915,54952916,54952917,54932918,54932919,-3132920,5211292  1,52122922,52132923,52142924,52152925,52162926,52212927,52222928,52232929,52232930,52232931,52232932,-3132933,52312934,52322935,52332936,52342  937,12322938,53152939,52142940,52232941,52232942,-3132943,52412944,524  22945,52432946,52442947,52452948,52492949,52492950,31122951,-3132952,52  242953,52242954,52242955,-3132956,54962957,54992958,54992959,54992960,5  4992961,54992962,54992963,54992964,54992965 |
| **5. Technology and Administration**  **·science, research, engineering, computer technology (including managers)**  **·office-based work: professional, managerial, administrative or general office/clerical**  Coding:  -3133017,-3133018,11373019,21113020,21123021,21123022,21133023,21133024  ,24233025,23213026,23223027,23223028,23293029,23113030,23293031,-313303  2,31113033,31193034,24513035,24523036,-3133037,21213038,21213039,212230  40,21233041,21243042,21253043,21263044,21273045,21283046,21293047,-3133  048,31123049,31133050,31143051,31153052,52493053,52493054,31193055,-313  3056,11363057,21313058,21323059,31313060,31323061,52443062,52453063,-3133198,-3133199,11113200,11133201,24413202,35613203,41113204,41123205,  41133206,35623207,35633208,35643209,-3133210,11123211,11143212,35623213  ,35633214,35643215,41143216,35623217,35633218,35643219,-3133220,1131322  1,11323222,11333223,11343224,11353225,12223226,12253227,11373228,11413229,11423230,12313231,35623232,35633233,35643234,-3133235,35413236,35423  237,35433238,34333239,41213240,41223241,72113242,72123243,41373244,35443245,35623246,35633247,35643248,-3133249,11513250,11523251,41213252,412  23253,41233254,41323255,41313256,35313257,35323258,35333259,35623260,35633261,35643262,-3133263,35313264,35323265,35333266,35343267,35353268,3  5363269,35373270,35393271,41313272,35623273,35633274,35643275,-3133276,  24113277,24193278,35203279,42123280,24213281,24223282,24233283,35623284,35633285,35643286,-3133287,24313288,24323289,24333290,24343291,312132  92,31223293,35623294,35633295,35643296,-3133297,42113298,42123299,42133  300,42143301,42153302,42163303,42173304,91493305,-3133306,41503307,4141  3308,41423309,41423310,92113311,92193312,-3133313,24513314,24523315,413  53316,41313317,41363318 |
